# Quantitative high-throughput assay to measure MC4R-induced intracellular calcium

**DOI:** 10.1530/JME-20-0285

**Published:** 2021-03-19

**Authors:** Shree Senthil Kumar, Marie-Louise Ward, Kathleen Grace Mountjoy

**Affiliations:** 1 Department of Physiology, Faculty of Medical and Health Sciences, University of Auckland, Auckland, New Zealand; 2 Department of Molecular Medicine and Pathology, Faculty of Medical and Health Sciences, University of Auckland, Auckland, New Zealand; 3 Maurice Wilkins Centre for Molecular Biodiscovery, Auckland, New Zealand

**Keywords:** melanocortin-4 receptor, cell signalling, calcium, G-protein-coupled receptor, high-throughput screening

## Abstract

The melanocortin-4 receptor (MC4R), a critical G-protein-coupled receptor (GPCR) regulating energy homeostasis, activates multiple signalling pathways, including mobilisation of intracellular calcium ([Ca^2+^]_i_). However, very little is known about the physiological significance of MC4R-induced [Ca^2+^]_i_ since few studies measure MC4R-induced [Ca^2+^]_i_. High-throughput, read-out assays for [Ca^2+^]_i_ have proven unreliable for overexpressed GPCRs like MC4R, which exhibit low sensitivity mobilising [Ca^2+^]_i_. Therefore, we developed, optimised, and validated a robust quantitative high-throughput assay using Fura-2 ratio-metric calcium dye and HEK293 cells stably transfected with MC4R. The quantitation enables direct comparisons between assays and even between different research laboratories. Assay conditions were optimised step-by-step to eliminate interference from stretch-activated receptor increases in [Ca^2+^]_i_ and to maximise ligand-activated MC4R-induced [Ca^2+^]_i_. Calcium imaging was performed using a PheraStar FS multi-well plate reader. Probenecid, included in the buffers to prevent extrusion of Fura-2 dye from cells, was found to interfere with the EGTA-chelation of calcium, required to determine R_min_ for quantitation of [Ca^2+^]_i_. Therefore, we developed a method to determine R_min_ in specific wells without probenecid, which was run in parallel with each assay. The validation of the assay was shown by reproducible α-melanocyte-stimulating hormone (α-MSH) concentration-dependent activation of the stably expressed human MC4R (hMC4R) and mouse MC4R (mMC4R), inducing increases in [Ca^2+^]_i_, for three independent experiments. This robust, reproducible, high-throughput assay that quantitatively measures MC4R-induced mobilisation of [Ca^2+^]_i_ *in vitro* has potential to advance the development of therapeutic drugs and understanding of MC4R signalling associated with human obesity.

## Introduction

Mutations in hMC4R are the single most common gene mutation associated with human obesity (Farooqi *et al.* 2000, Vaisse *et al.* 2000). For years, studies investigating native and mutant MC4R signalling have focused on MC4R-induced increases in cyclic AMP (cAMP) (Yeo *et al.* 2003), activation of CRE-reporter gene transcription (Wright *et al.* 2015), and phosphorylation of ERK (Daniels *et al.* 2003, Vongs *et al.* 2004, Chai *et al.* 2006, Paisdzior *et al.* 2020). Recently, hMC4R recruitment of β-arrestin was investigated, and a significant correlation was found between the efficacy of mutant MC4R-β-arrestin recruitment and BMI but not between cAMP and BMI (Lotta *et al.* 2019). A second study also showed mutant MC4R exhibit decreased, or increased, β-arrestin recruitment *in vitro* (Gillyard *et al.* 2019). Both studies indicate that MC4R-β-arrestin signalling is more closely associated with obesity compared with MC4R-associated cAMP, which emphasises a need to investigate MC4R signalling beyond cAMP. Measurement of [Ca^2+^]_i_ is not considered a reliable high-throughput read-out in an overexpression system (Westerink & Hondebrink 2010, Meijer *et al.* 2014, Gillyard *et al.* 2019). Recently, using microscopy analysis, Class V obesity-associated hMC4R variants (i.e. those that are obesity associated but appear similar to WT on cAMP assays and protein expression studies) were shown to mobilise [Ca^2+^]_i_ with similar efficacy to WT hMC4R (Sharma *et al.* 2020). However, application of this method is limited as it is neither of high throughput nor quantitative. A high-throughput nuclear factor of activated T cell (NFAT) luciferase reporter gene assay was recently used to show that Class V obesity-associated hMC4R variants exhibit impaired activation of phospholipase C-β (PLC) compared with WT hMC4R (Clement *et al.* 2018). It is assumed that this signalling is via PLC-induced increased inositol triphosphate and mobilisation of intracellular calcium. Because α-MSH-induced [Ca^2+^]_i_ in HEK293 cells is not associated with an increase in inositol triphosphate but is significantly attenuated by cholera toxin and therefore dependent on Gαs (Mountjoy *et al.* 2001), the signalling pathway monitored by NFAT luciferase reporter in HEK293 cells may or may not involve α-MSH-induced [Ca^2+^]_i_. Nevertheless, while the intracellular signalling pathway responsible for α-MSH-induced [Ca^2+^]_i_ in HEK293 cells remains unknown, there is a need to develop a high-throughput assay to specifically measure MC4R-induced mobilisation of [Ca^2+^]_i_ *in vitro*.

High-throughput GPCR signalling assays, comprising cell monolayers in 96-well plates with automated capturing of a signalling response (Kurko *et al.* 2009, Valentine & Tigyi 2012), can have associated problems. The first problem is to eliminate ‘stretch-receptor-activated mobilisation of [Ca^2+^]_i_ which interferes with ligand-induced mobilisation of [Ca^2+^]_i_ and contributes to assay variability. Stretch-receptor responses are triggered by turbulence resulting from injecting ligands into media covering the cell monolayer and/or sudden movement of the plate (Demer *et al.* 1993, Naruse & Sokabe 1993, Sigurdson *et al.* 1993, Rosales *et al.* 1997, Tong *et al.* 1999, Hayakawa *et al.* 2008, Heusinkveld & Westerink 2011, Meijer *et al.* 2014). Secondly, there is potential for non-homogeneous distribution of the calcium-binding dye in a cell monolayer. Accumulation of dye in multiple, discrete subcellular compartments blunts ligand-induced calcium transients (Malgaroli *et al.* 1987). Thirdly, plate-reader-based methods have low temporal and spatial resolution resulting in low sensitivity for measuring highly dynamic and transient changes in [Ca^2+^]_i_ (Meijer *et al.* 2014). Fourthly, most high-throughput assays measuring calcium transients use single-wavelength fluorescent dyes such as Fluo-4 that are not quantitative and are subject to interference from a number of factors (Kurko *et al.* 2009, Galaz-Montoya *et al.* 2017). Comparisons of transient changes in [Ca^2+^]_i_ are only possible within an assay using single-wavelength calcium indicators.

Dual-wavelength calcium indicators such as Fura-2 undergo a spectral shift proportional to [Ca^2+^]_i_ and are not subject to interference seen with single-wavelength calcium dyes (Paredes *et al.* 2008). Peak-emitted fluorescence of Fura-2 at 510 nm shifts with excitation wavelength from 340 nm in the calcium-bound state to 380 nm in the calcium-free state. Fura-2-Ca^2+^ is quantitated by computing the ratio of fluorescence at two wavelengths, independent of dye concentration in the assay (Grynkiewicz *et al.* 1985, Malgaroli *et al.* 1987). Therefore, average cytosolic-free calcium released during an assay can be quantitated with Fura-2, providing that minimum (R_min_) and maximum (R_max_) Ca^2+^ can be determined.

Previously, using cell suspensions and a manual assay with Fura-2, carbachol was shown to activate endogenous M3 muscarinic acetylcholine receptor (M3-AChR) in HEK293 cells increasing [Ca^2+^]_i_, while α-melanocyte-stimulating hormone (α-MSH) activated stably expressed mMC4R stimulating, a reduced [Ca^2+^]_i_ response (~three-fold lower) compared with carbachol-stimulated M3-AChR response (Mountjoy *et al.* 2001). Traditional FLIPR assays, commonly used to measure relative [Ca^2+^]_i_ concentrations, appear not to have been used to demonstrate MC4R coupling to [Ca^2+^]_i_. This may be due to the FLIPR assay not having the sensitivity required to detect relatively low efficacious MC4R-induced [Ca^2+^]_i_ responses (Mountjoy *et al.* 2001, Newman *et al.* 2006, Sharma *et al.* 2020). Here, we developed and validated a high-throughput assay using Fura-2 to quantitate [Ca^2+^]_i_ responses for stably expressed hMC4R and mMC4R in HEK293 cells. We evaluated assay conditions in a step-by-step manner. Finally, we validated the high-throughput quantitative [Ca^2+^]_i_ assays for α-MSH-concentration-dependent activation of hMC4R and mMC4R and carbachol-concentration-dependent activation of M3-AChR was used as a positive control.

## Materials and methods

### Reagents

DMEM, newborn calf serum (NCS), penicillin and streptomycin (P/S), Fugene 6 transfection reagent, and Geneticin (G418) were purchased from Invitrogen. Poly-L-lysine (PLL), ionomycin calcium salt, ethylene glycol-bis (β-aminoethyl ether)-N,N,N′,N′-tetraacetic acid (EGTA) and carbamylcholine chloride (carbachol) were purchased from Sigma-Aldrich. Fura-2/AM, pluronic acid F-127, probenecid, and HBSS were purchased from Life Technologies. α-MSH was purchased from Bachem, Switzerland. Corning 96-well black, clear-bottom polystyrene, tissue-culture-treated microplates were purchased from Invitro Technologies.

### Cells and plasmids

The human embryonic kidney (HEK293) cells used in this study were originally sourced from ATCC and authenticated in this laboratory. For the SNPs tested, they are 100% identical to ATCC clone CRL-12013. The hMC4R and mMC4R cloned in pcDNA3.1 and HEK293 cells stably expressing hMC4R or mMC4R were previously developed (Mountjoy *et al.* 1994, Kay *et al.* 2015).

### Buffers

The buffers used were: CLB-LG (132 mM NaCl, 5 mM KCl, 5 mM Na_2_HPO_4_, 1.2 mM NaH_2_PO_4_·H_2_O, 1 mM CaCl_2_·2H_2_O, 0.8 mM MgCl_2_·6H_2_O, 1 mM Glucose); CLB-HG (132 mM NaCl, 5 mM KCl, 5 mM Na_2_HPO_4_, 1.2 mM NaH_2_PO_4_·H_2_O, 1 mM CaCl_2_·2H_2_O, 0.8 mM MgCl_2_·6H_2_O, 10 mM Glucose); Ca^2+^-free buffer-HG (132 mM NaCl, 5 mM KCl, 5 mM Na_2_HPO_4_, 1.2 mM NaH_2_PO_4_·H_2_O, 0.8 mM MgCl_2_·6H_2_O, 10 mM Glucose); Ca^2+^-free buffer–Ward (118 mM NaCl, 6 mM KCl, 1.18 mM MgSO_4_, 1.18 mM KH_2_PO_4_, 24.8 mM NaHCO_3_, 10 mM glucose) and Ca^2+^-free buffer Ion Optix (10 mM NaCl, 150 mM KCl, 3 mM MgCl_2_, 10 mM HEPES).

### Cell culture

HEK293 cells were grown in DMEM supplemented with 10% (v/v) NCS and 1% (v/v) P/S at 37 °C under 5% CO_2_. The hMC4R or mMC4R stably transfected HEK293 cells were maintained in the presence of 500 μg/mL G418.

### Experimental design

**Figure 1:**
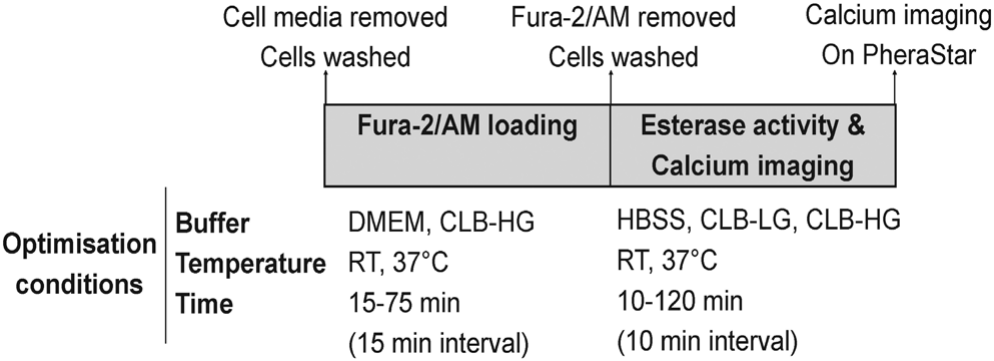
Schematic showing the experimental parameters optimised for measuring [Ca^2+^]_i_ in HEK293 cell monolayers.

First, the speed and volume of vehicle injection onto HEK293 cell monolayer were optimised to eliminate stretch-receptor activation of [Ca^2+^]_i_. Secondly, the dye-loading and esterase-activity buffers, incubation times, and incubation temperatures were optimised in a step-by-step manner (Fig. 1) for use with Fura-2/AM, a membrane-permeable dye (Grynkiewicz *et al.* 1985). Each step was optimised to eliminate vehicle-induced stretch-receptor activation and maximise carbachol-induced M3-AChR mobilisation of [Ca^2+^]_i_ (positive control). Thirdly, the method was validated for reproducible quantitative analysis of carbachol-induced M3-AChR mobilisation of [Ca^2+^]_i_ and α-MSH-induced hMC4R and mMC4R mobilisation of [Ca^2+^]_i_.

### Optimisation of injection speed and injection volume to prevent stretch-receptor-activated [Ca**^2+^**]_i_ in HEK293 cell monolayer

HEK293 cells (4.5 × 10^4^ cells/0.2 mL/well) were plated in PLL coated 96-well plates. After 48 h, cells (~1.2 × 10^5^ cells/well) were washed once with 0.2 mL DMEM/25 mM HEPES containing 2.5 mM probenecid. Cells were then loaded with 100 µL of this media containing Fura-2/AM (1 µg/µL) premixed with 20 % pluronic acid F-127 in DMSO (1:1), and added to media to give 2 µM Fura-2/AM final concentration. Cells were incubated at RT for 60 min in the dark (wrapped in foil). Loading buffer was removed, and cells washed once with 0.2 mL CLB-HG containing 2.5 mM probenecid. CLB-HG containing 2.5 mM probenecid (0.29 mL) was added to each well, and the plate incubated at 37°C for 90 min inside the PheraStar FS. During this incubation, Fura-2/AM was de-esterified to Fura-2. To test for stretch-receptor activation, vehicle (CLB-HG) ranging from 10 to 50 µL was injected into each well with speeds ranging from 100 to 430 µL/s.

### Optimisation of buffers, incubation temperatures, and incubation times for Fura-2/AM loading and Fura-2/AM esterase cleavage plus calcium imaging

Buffers, incubation times, and temperatures for Fura-2/AM loading and esterase cleavage of Fura-2/AM to release Fura-2 were optimised as outlined in Fig. 1.

### Development of quantitative [Ca**^2+^**]_i_ assay for use with cell monolayers in 96-well plate

[Ca^2+^]_i_ was calculated using the f340/f380 ratio and the Grynkiewicz * et al.* equation with K_d_ = 224 (Grynkiewicz *et al.* 1985). To determine R_min_ and R_max_ values, EGTA (10 µL of 165 mM stock in water) was injected at 30 s to chelate calcium, and ionomycin (10 µL of 0.33 mM stock in DMSO) was injected at 30 s to increase extracellular calcium flux across the plasma membrane and saturate Fura-2 binding.

### Development of quantitative [Ca**^2+^**]_i_ assay for use with cell suspensions in 96-well plate

Confluent cells grown in T75 tissue culture flasks were washed and detached using versene, diluted in DMEM/25 mM HEPES containing 10% NCS, and pelleted by centrifugation at 950 ***g*** for 5 min. The cell pellet was washed with DMEM/25 mM HEPES, pelleted again by centrifugation, and then the cells were resuspended in DMEM/25 mM HEPES. A 1:1 mix of Fura-2/AM with pluronic acid in DMSO was added to the cell suspension to give 2 µM Fura-2/AM final concentration. Cells were incubated at RT for 60 min in dark with gentle mixing by manually rotating the tube. Cells were pelleted, washed once with CLB-HG containing 1 mM probenecid and pelleted again. The cell pellet was gently resuspended in CLB-HG containing 1 mM probenecid to give 8.5 × 10^5^ cells/mL and cells incubated at 37°C for 80 min in dark with gentle mixing. Cells were then pipetted into PLL coated 96-well plates (~1.8 × 10^5^ cells/0.21 mL/well) for calcium imaging in the PheraStar FS.

The well volume (0.21–0.29 mL), injection volume (10–30 µL) and injection speed (100–150 µL/s) for the addition of vehicle or ligand was optimised for cell suspensions. Baseline, vehicle, and α-MSH (10^−6^–10^−10^ M) stimulated 340/380 ratios were monitored and [Ca^2+^]_i_ was quantitated following the protocol outlined for quantitation of [Ca^2+^]_i_ in cell monolayers.

### Calcium imaging

**Figure 2:**
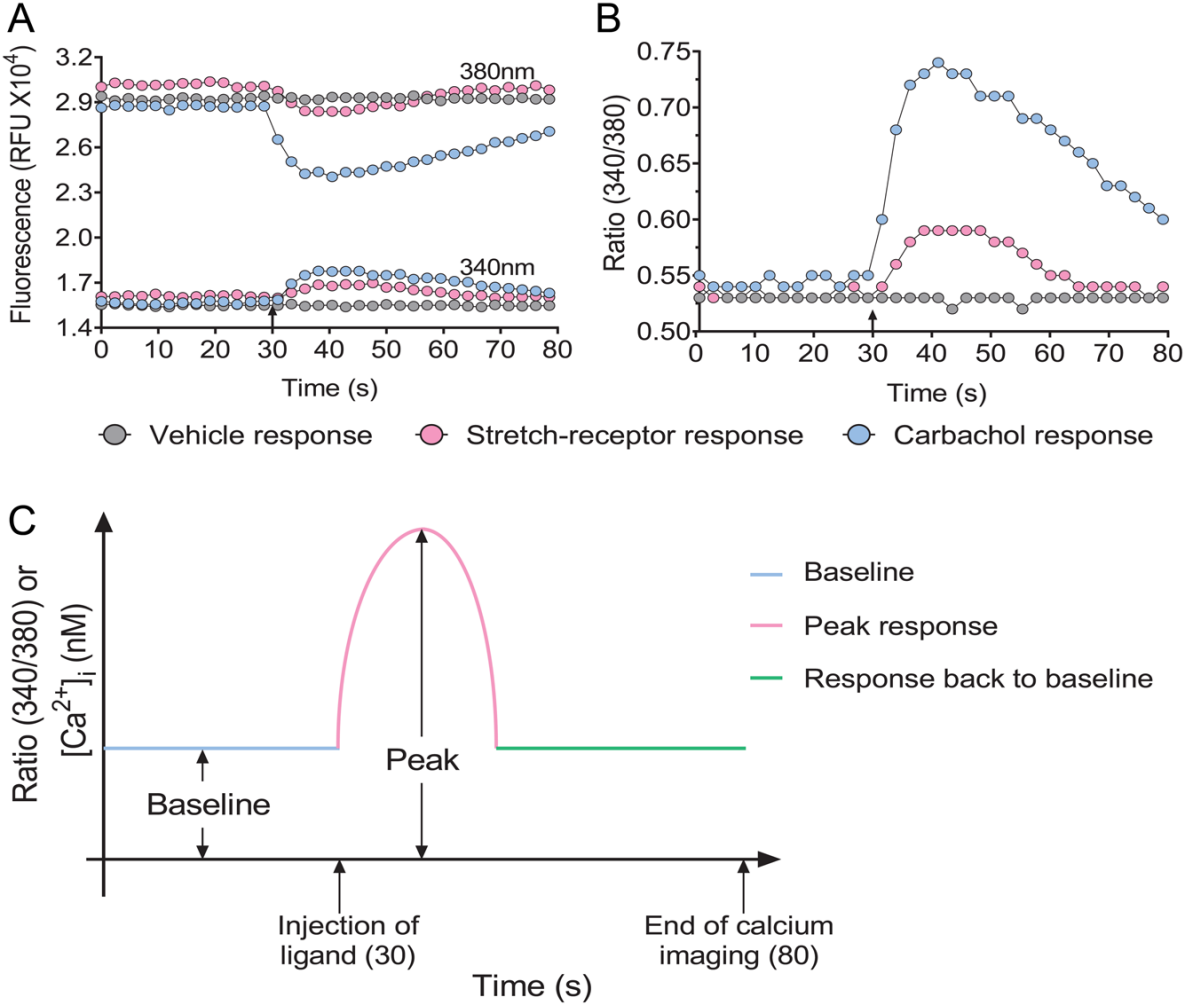
Representative trace of fluorescence kinetic data from single-wells showing stretch-receptor, vehicle and agonist-induced [Ca^2+^]_i_ in HEK293 cell monolayers. (A) The raw fluorescence (RFU) at 340 nm and 380 nm for stretch-receptor, vehicle and agonist-induced calcium responses. (B) The fluorescence ratio (340/380) corresponding to the raw fluorescence values for stretch-receptor, vehicle and agonist-induced calcium responses. (C) Schematic showing how baseline and peak response for measurement of [Ca^2+^]_i_ is calculated. Arrows indicate the time (30 s) that the vehicle/agonist was injected and the time calcium imaging was finished (80 s).

The 96-well plates with cells were imaged using PheraStar FS (BMG LABTECH) at 37°C. Baseline fluorescence intensity for each well was recorded for 30 s using 340/380 nm excitation and 510 nm emission wavelengths (Fig. 2A). Vehicle/ligand was injected at 30 s, and the 340/380 ratio (Fig. 2B) was monitored post-injection for 50 s. The peak 340/380 ratio was calculated by subtracting the baseline 340/380 ratio from the maximum 340/380 ratio (Fig. 2C). An increase or decrease in the 340/380 ratio reflects an increase or decrease in calcium signal, respectively. The basal and agonist-stimulated carbachol response was used as an indicator for the optimal conditions.

### PheraStar FS setup

The PheraStar FS plate reader was set up for imaging calcium with a settling time of 0.5 s before measurement of each well, and with a bi-directional horizontal reading direction to avoid activation of stretch-receptor calcium channels. The number of flashes (10) per well was set to average to one intensity value per well to improve accuracy. The kinetic window was set to 80 s, and the plate was set at the bottom optic to measure fluorescence. The focus and gain were adjusted prior to running each assay. The focal height adjustment of the optical system ensures best signal-to-noise ratio for the volume in every well. The gain adjustment optimises signal amplification so that maximum sensitivity was achieved.

### Statistics

GraphPad Prism 7.0 software (GraphPad Software Incorporated) was used to generate graphs and perform statistical analysis. Carbachol- and α-MSH-concentration-response curves were fitted to raw [Ca^2+^]_i_ data to obtain EC_50_ values from three independent experiments. The quantitative [Ca^2+^]_i_ data from three independent experiments were then pooled for comparison of maximum, minimum, or EC_50_ values between carbachol-induced and α-MSH-induced [Ca^2+^]_i_.

## Results

### Ten microlitres injection at a speed of 100 µL/s into 290 µL buffer covering a HEK293 cell monolayer eliminates stretch-receptor Ca**^2+^** response

We found that a 10 μL injected at 100 μL/s into a well containing 290 μL buffer consistently eliminated stretch-receptor activated [Ca^2+^]_i_ response in HEK293 cell monolayers (Supplementary Table 1, see section on supplementary materials given at the end of this article).

### Dulbecco’s modified Eagle medium (DMEM) is the optimal buffer for Fura-2/AM loading

**Figure 3:**
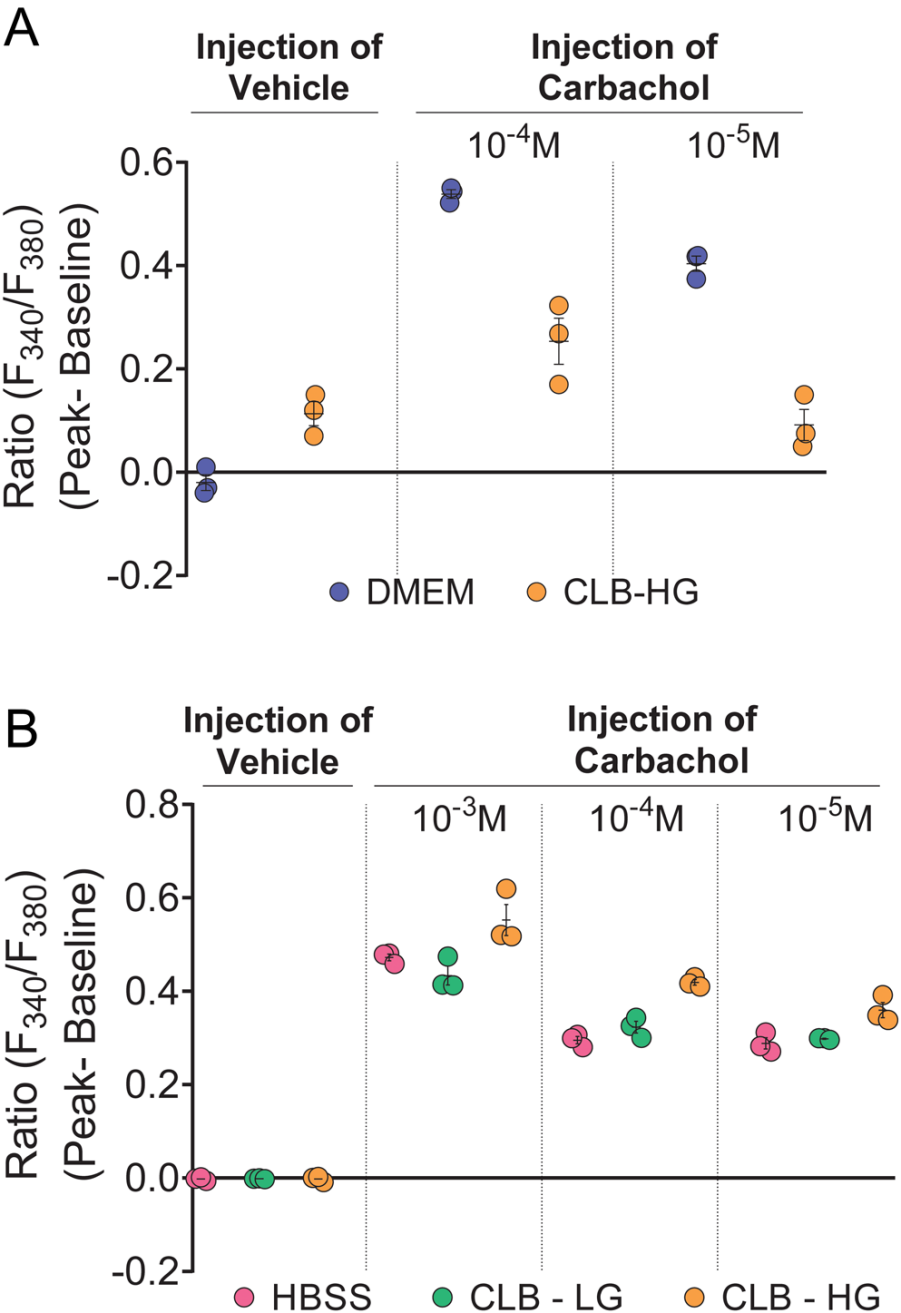
Optimal buffer for Fura-2/AM loading is DMEM and optimal buffer for esterase cleavage and calcium imaging is CLB-HG. (A) DMEM and CLB-HG were compared for Fura-2/AM loading, and the cells were tested for vehicle induced stretch-activated calcium response and carbachol (10^−4^ and 10^−5^ M)-induced calcium signal; (B) HBSS, CLB-LG, and CLB-HG were compared as buffers for esterase cleavage and calcium imaging. The cells were tested for vehicle-induced stretch-activated calcium response and carbachol (10^−3^, 10^−4^ and 10^−5^ M) -induced calcium signal. Data shown as mean ± s.e.m. are representative of two experiments. Data for all experiments are shown in Supplementary Fig. 1A, B, C, D, E, F and G.

Fura-2/AM loading into HEK293 cells was compared between buffers, DMEM and calcium loading buffer-high glucose (CLB-HG). Data from two independent experiments showed that DMEM was optimal for Fura-2/AM loading. In contrast with CLB-HG, there was no vehicle-induced activation of stretch-receptor calcium response in the presence of DMEM. Furthermore, two doses of carbachol (10^−4^ and 10^−5^ M) in two independent experiments consistently induced higher calcium signal with DMEM compared with CLB-HG (Fig. 3A and Supplementary Fig. 1A, B, C).

### CLB-HG is the optimal buffer for esterase cleavage of Fura-2/AM and calcium imaging

Following Fura-2/AM loading in DMEM, buffers: hanks balanced salt solution (HBSS), calcium loading buffer-low glucose (CLB-LG), and CLB-HG were compared for optimal esterase cleavage of Fura-2/AM. Data from two independent experiments consistently showed no vehicle-induced stretch-activated calcium response with any of these buffers. However, three different doses of carbachol (10^−3^, 10^−4^ and 10^−5^ M) in two independent experiments, consistently induced higher calcium signal in the presence of CLB-HG compared with HBSS or CLB-LG (Fig. 3B and Supplementary Fig. 1D, E, F, G).

### Room temperature (RT) is optimal for Fura-2/AM loading

**Figure 4:**
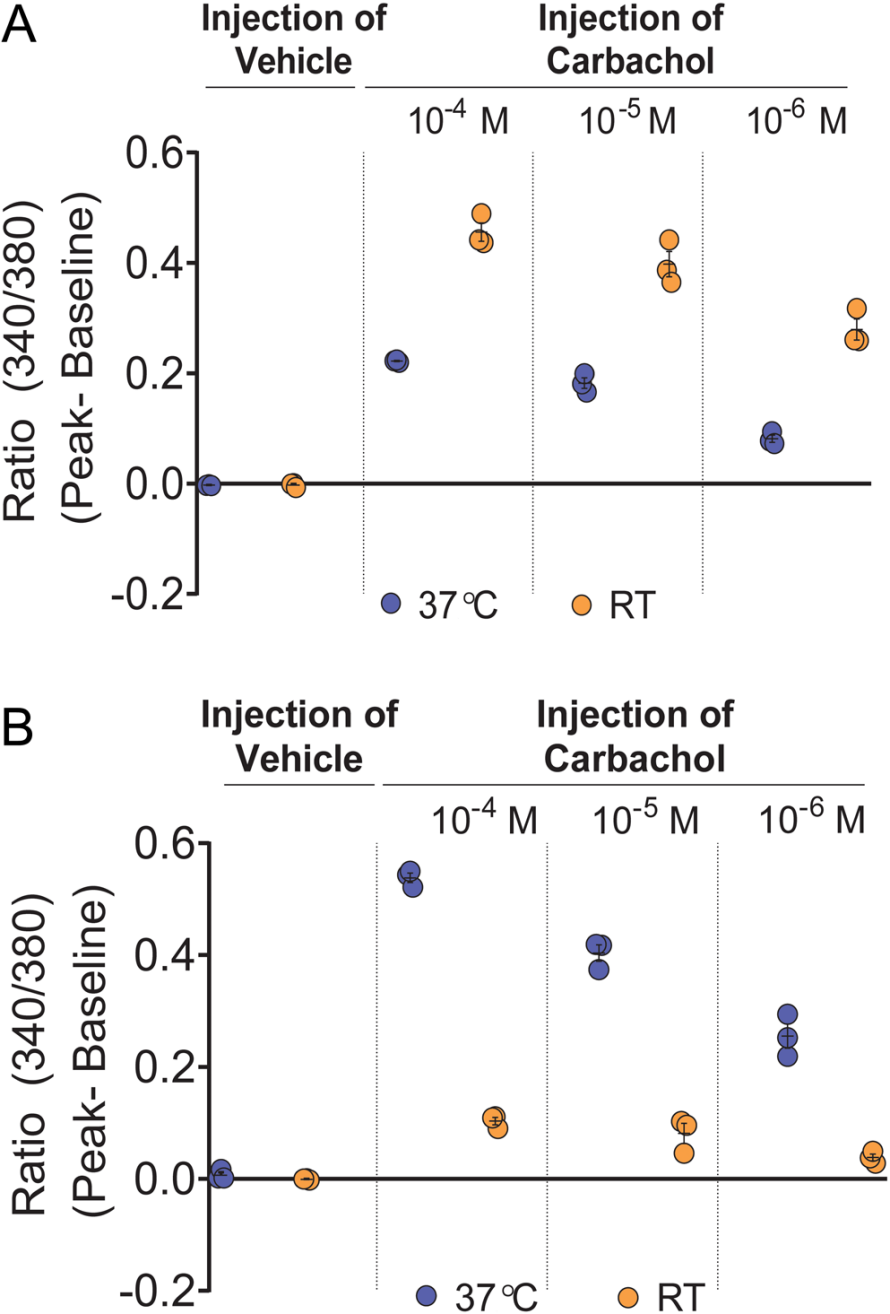
Optimal temperature for Fura-2/AM loading is RT and the optimal temperature for esterase cleavage of Fura-2/AM is 37°C. (A) RT and 37°C were compared for Fura-2/AM loading and the cells were tested for vehicle-induced stretch-activated calcium response and carbachol (10^−4^ M, 10^−5^ M and 10^−6^ M) -induced calcium signal; (B) RT and 37°C were compared for esterase cleavage of Fura-2/AM and the cells were tested for vehicle-induced stretch-activated calcium response and carbachol (10^−4^, 10^−5^ and 10^−6^ M) -induced calcium signal. Data shown as mean ± s.e.m. are representative of two experiments. Data for all experiments are shown in Supplementary Fig. 2A, B, C, D, E, F, G and H.

We compared Fura-2/AM loading in DMEM between incubation temperatures, RT and 37°C. Data from two independent experiments consistently showed no vehicle-induced stretch-activated calcium response at either RT or 37°C. Our data confirmed previous findings that dye loaded into cell monolayers at 37°C blunts ligand-induced mobilisation of [Ca^2+^]_i_. Three doses of carbachol (10^−4^, 10^−5^ and 10^−6^ M) consistently induced higher calcium signal when Fura-2/AM loading was performed at RT compared with 37°C (Fig. 4A and Supplementary Fig. 2A, B, C, D).

### Optimal temperature for esterase cleavage of Fura-2/AM is 37°C

Data from two independent experiments consistently showed no vehicle-induced stretch-activated calcium response at either RT or 37°C. However, three doses of carbachol (10^−4^, 10^−5^ and 10^−6^ M) consistently induced higher calcium signal when Fura-2/AM esterase cleavage was performed at 37°C, compared with RT (Fig. 4B and Supplementary Fig. 2E, F, G, H).

### Optimal incubation time for Fura-2/AM loading is 60 min

**Figure 5:**
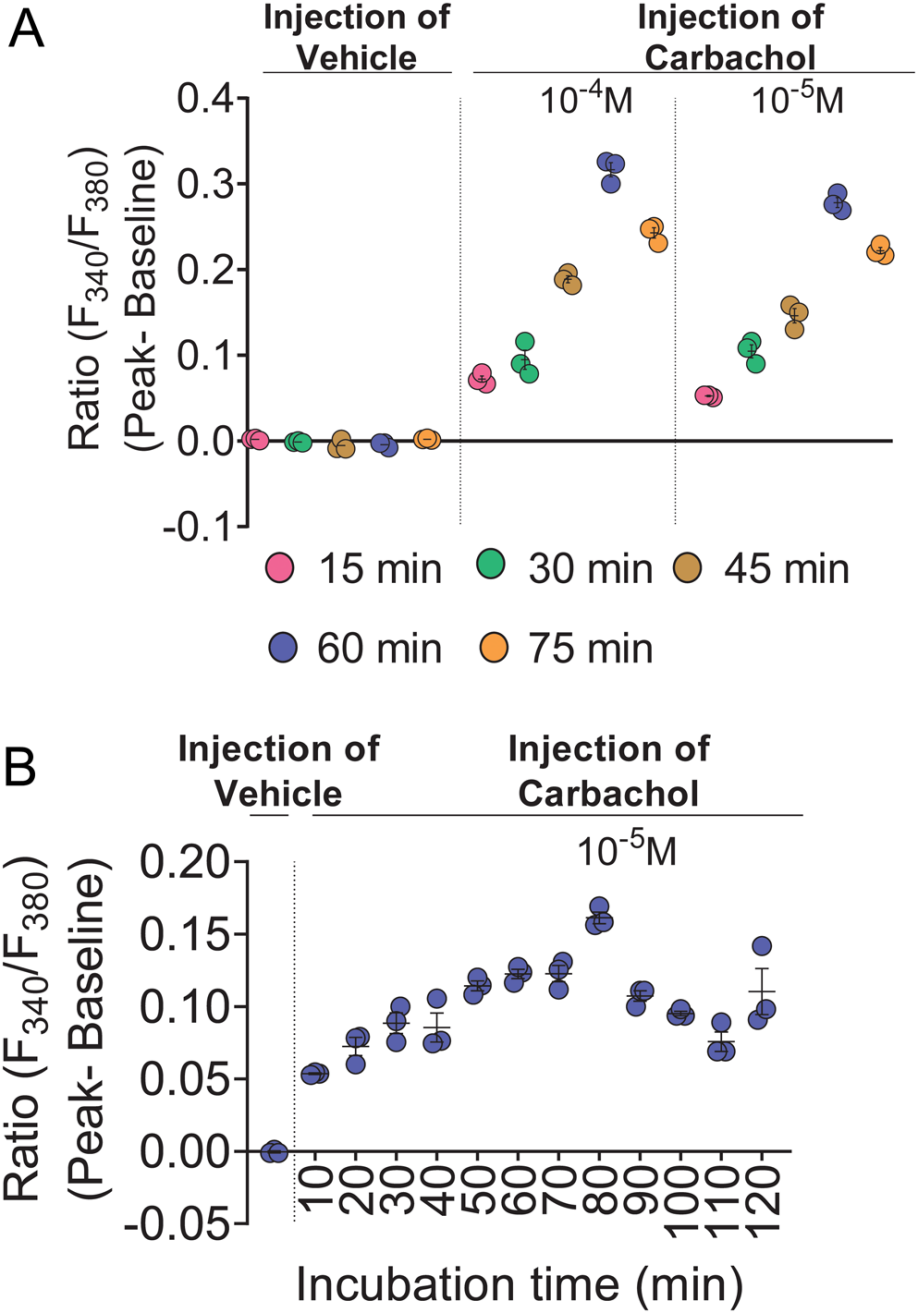
Optimal incubation time for Fura-2/AM loading is 60 min and optimal incubation time for cytosolic esterase cleavage of Fura-2/AM is 80 min. (A) Incubation times ranging from 15 to 75 min were compared for Fura-2/AM loading and then the cells were tested for vehicle-induced stretch-activated calcium response and carbachol (10^−4^ and 10^−5^ M) -induced calcium signal. (B) Incubation times ranging from 10 to 120 min were compared for esterase cleavage of Fura-2/AM and then the cells were tested for vehicle-induced stretch-activated calcium response and carbachol (10^−5^ M) -induced calcium signal. Data shown as mean ± s.e.m. are representative of three experiments. Data for all experiments are shown in Supplementary Fig. 3A, B, C, D, E, F and G.

Fura-2/AM loading in DMEM at RT was compared at 15 min intervals for incubation times from 15 to 75 min. Data from three independent experiments consistently showed no vehicle-induced stretch-activated calcium response for Fura-2/AM loading performed at any of these incubation times. However, two doses of carbachol (10^−4^ and 10^−5^ M) consistently induced higher calcium signal when Fura-2/AM loading was conducted at RT for 60 min compared with other incubation times (Fig. 5A and Supplementary Fig. 3A, B, C).

### Optimal incubation time for esterase cleavage of Fura-2/AM is 80 min

Endogenous esterase cleavage of Fura-2/AM following loading in DMEM at RT for 60 min was compared at 10 min intervals from 10 to 120 min with incubation in CLB-HG at 37°C. Data from three independent experiments consistently showed no vehicle-induced stretch-activated calcium response when cells were incubated for 90 min compared with the other incubation times. Carbachol (10^−5^ M) consistently induced higher calcium signal when cells were incubated for 80 min compared with other times (Fig. 5B and Supplementary Fig. 3D, E, F, G).

### Validation that addition of pluronic acid enhances Fura-2/AM loading in HEK293 cell monolayers

**Figure 6:**
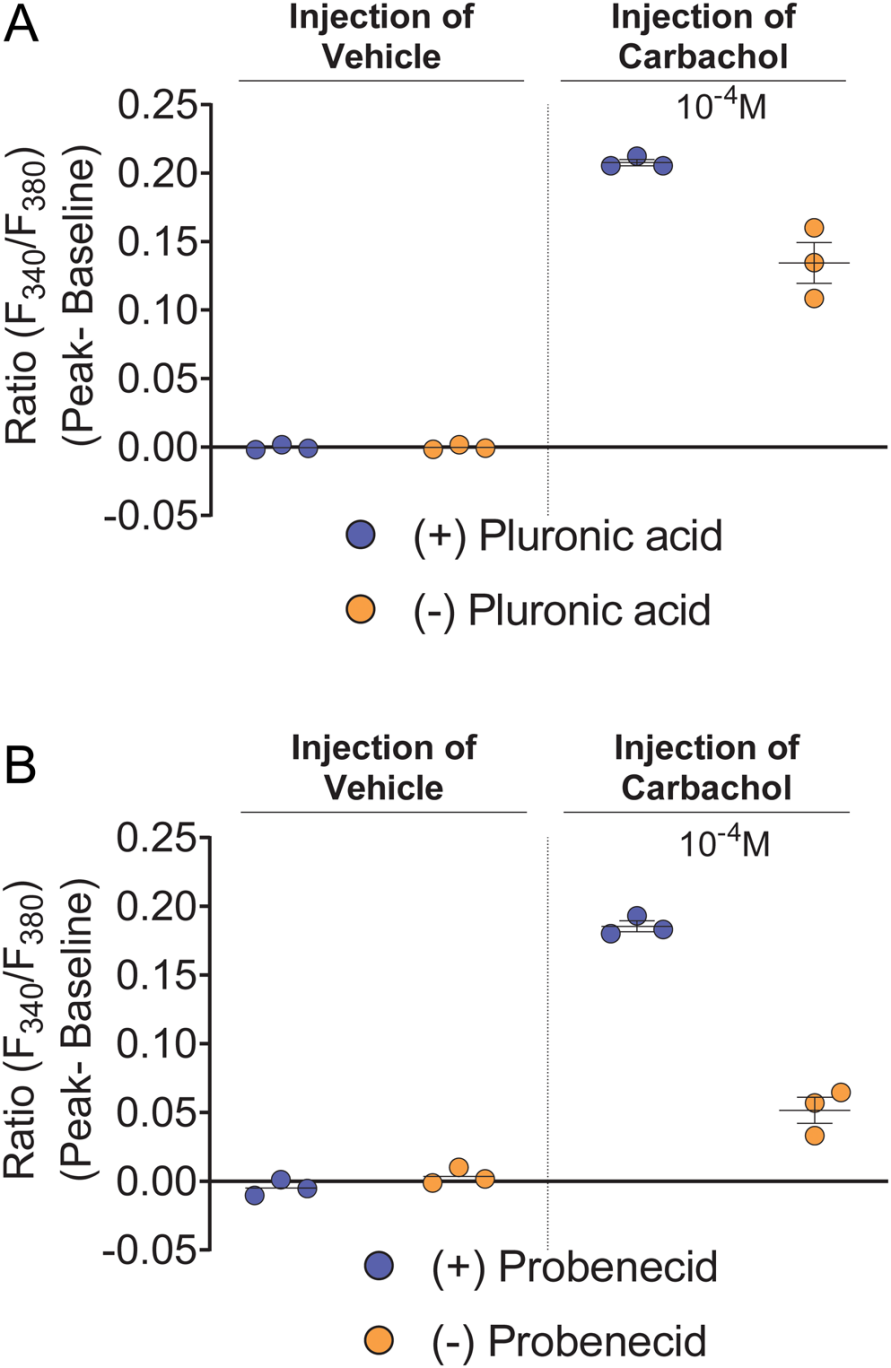
Pluronic acid and probenecid enhanced carbachol-activated M3-AChR-induced calcium signal. (A) Fura-2/AM was loaded in DMEM the presence or absence of pluronic acid and the cells were then tested for vehicle-induced stretch-activated calcium response and carbachol (10^−4^ M) -induced calcium signal. (B) Probenecid (2.5 mM) was present or not present during Fura-2/AM loading and esterase cleavage and then the cells were then tested for vehicle-induced stretch-activated calcium response and carbachol (10^−4^ M) -induced calcium signal. Data shown as mean ± s.e.m. are representative of three independent experiments. Data for all experiments are shown in Supplementary Fig. 4A, B, C and D.

Pluronic acid is a surfactant polyol that is frequently used to increase the incorporation of Fura-2/AM into cells (Krylova & Pohl 2004). Here, we validated that pluronic acid enhances Fura-2/AM loading into HEK293 cell monolayers. Data from three independent experiments consistently showed no vehicle-induced stretch-activated calcium response in the absence or presence of pluronic acid in DMEM. Carbachol (10^−4^ M) consistently induced higher calcium signal when Fura-2/AM loading was conducted in the presence of pluronic acid compared with no pluronic acid (Fig. 6A and Supplementary Fig. 4A, C).

### Validation that addition of probenecid during Fura-2/AM loading and esterase-cleavage enhances cellular retention of Fura-2/AM

Probenecid is an organic anion pump inhibitor that is used to inhibit the extrusion of Fura-2/AM into the extracellular environment (Di Virgilio *et al.* 1988, Di Virgilio *et al.* 1990, Roe *et al.* 1990, Li *et al.* 2008, O’Connor & Silver 2013). Here, we validated that probenecid (2.5 mM) retains Fura-2/AM and Fura-2 in HEK293 cell monolayers. Data from three independent experiments consistently showed no vehicle-induced stretch-activated calcium response in the absence or presence of probenecid. Carbachol (10^−4^ M) consistently induced higher calcium signal when probenecid was included both in Fura-2/AM loading buffer (DMEM) and Fura-2/AM esterase cleavage buffer (CLB-HG) (Fig. 6B and Supplementary Fig. 4B, D).

### Development of a method to determine R**_min_** and R**_max_** for a quantitative [Ca**^2+^**]_i_ assay using HEK293 cell monolayers

Fura-2[Ca^2+^]_i_ is quantitated by computing the ratio of fluorescence at two wavelengths and determining R_min_ and R_max_ [Ca^2+^]_i_ (Grynkiewicz *et al.* 1985, Malgaroli *et al.* 1987). A decrease in basal calcium signal is observed when EGTA chelates calcium and this is used to determine R_min_. Surprisingly, EGTA injections did not reduce basal calcium signal in cell monolayers using the optimised assay conditions. EGTA was previously successfully used for determining R_min_ in the absence of pluronic acid and probenecid using HEK293 cell suspensions (Mountjoy *et al.* 2001). Therefore, EGTA chelation of calcium was assessed in the presence and absence of both pluronic acid and probenecid. Here, we show that probenecid (0.5–2.5 mM), but not pluronic acid, interferes with EGTA chelation of calcium. We did not observe the expected decrease in basal calcium signal, thus preventing the determination of R_min_ (Supplementary Fig. 5). We questioned whether the probenecid interference of EGTA-calcium chelation could be due to esterase activity buffer used. Previous studies have used calcium-free buffers for EGTA chelation of calcium to determine R_min_ (Sauve *et al.* 1990, Ward *et al.* 2003), one with reduced probenecid (1 mM) (Zhang *et al.* 2008). We determined that 1 mM probenecid worked as efficiently as 2.5 mM for Fura-2 retention since there was no blunting of carbachol-stimulated calcium signal with 1 mM probenecid (Supplementary Fig. 6). However, addition of 1 mM probenecid to CLB-HG, Ca^2+^-free buffer-HG, Ca^2+^-free buffer-Ward, or Ca^2+^-free buffer-Ion Optix buffer interfered with EGTA chelation of calcium (Supplementary Fig. 7).

**Figure 7:**
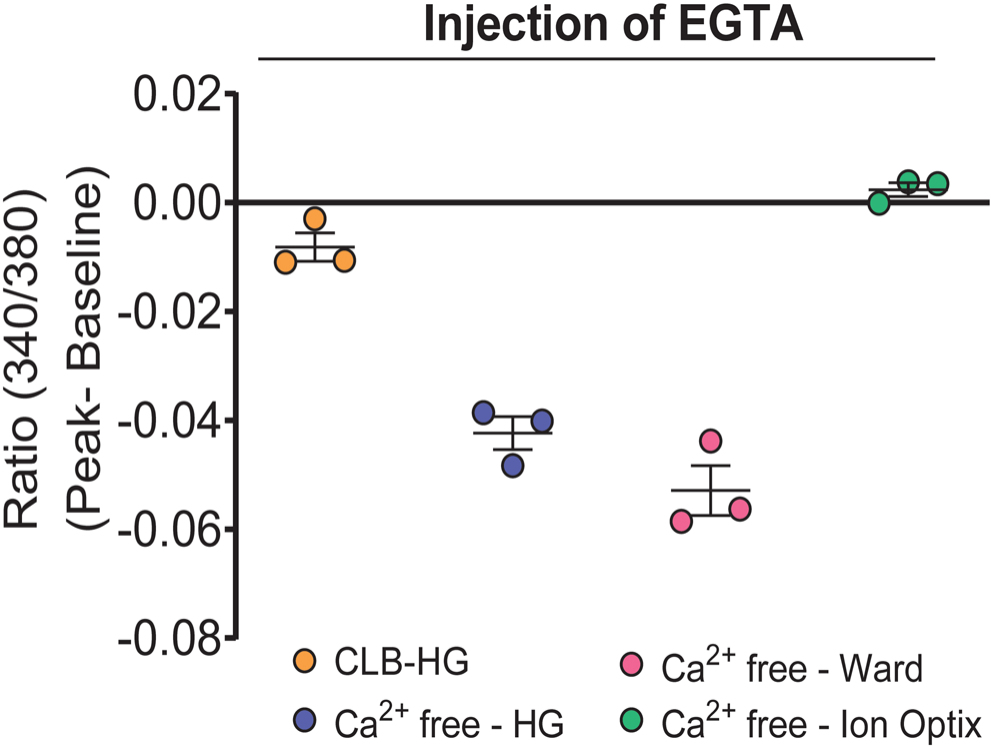
EGTA sequestration of calcium during fura-2/AM loading enabled use of EGTA injection to chelate calcium for determination of R_min_ for quantitation [Ca^2+^]_i_. The cells were co-loaded with Fura-2/AM and 10 µM EGTA in DMEM. Following this, probenecid (1 mM) was included in the CLB-HG buffer, or one of three different Ca^2+^-free buffers, for esterase cleavage and calcium imaging. EGTA (5.5 mM) was injected into these buffers during calcium imaging to chelate [Ca^2+^]_i_ for determination of R_min_. Data shown as mean ± s.e.m. are representative of three experiments. Data for all experiments are shown in Supplementary Fig. 8.

It is known that cells kept in EGTA rapidly become depleted of intracellular calcium stores (Hoth & Penner 1992, Struk *et al.* 1998). EGTA then prevents the refilling of these stores by acting as a scavenger. Therefore, to overcome probenecid interference of EGTA chelation of calcium, we tested whether R_min_ could be determined using EGTA in the absence of probenecid, using specific R_min_ wells in each assay. These had Fura-2/AM loaded in DMEM (-probenecid) in the presence of EGTA (10 µM final concentration). Probenecid (1 mM) was then included in different esterase activity buffers; the buffer containing calcium, CLB-HG (Mountjoy *et al.* 2001), and three different buffers with no calcium, Ca^2+^-free-HG, Ca^2+^-free buffer-Ward (Ward *et al.* 2003), and Ca^2+^-free buffer-Ion Optix (https://www.ionoptix.com/resource/loading-fura-2-into-cardiomyocytes/), and then EGTA chelation of calcium was tested. Three independent experiments consistently showed that injection of EGTA (5.5 mM final concentration) into these wells during imaging chelated calcium in the presence of either Ca^2+^-free-HG buffer or Ca^2+^-free-Ward buffer (Fig. 7 and Supplementary Fig. 8). We chose to use the Ca^2+^-free-HG buffer to determine R_min_ because this buffer was similar to the CLB-HG used when measuring ligand-stimulated increases in (Ca^2+^)_i_.

Ionomycin, an ionophore that facilitates the transfer of calcium and floods the cells with extracellular calcium, is used to measure R_max_. There was a rapid increase in calcium signal following ionomycin injection in the presence or absence of probenecid.

### Validation for the assay developed here to quantitate carbachol-induced endogenous M3-AChR mobilisation of [Ca**^2+^**]_i_ in HEK293 cell monolayer

**Figure 8:**
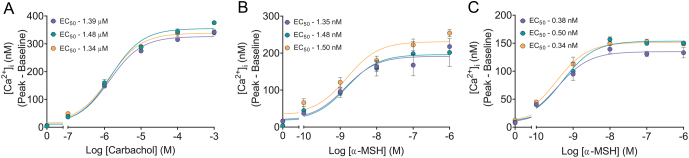
Validation of high-throughput quantitative ligand-activated GPCR-induced [Ca^2+^]_i_ assay using cell monolayers or cell suspensions. (A) Carbachol concentration-dependent activation of M3-AChR-induced [Ca^2+^]_i_ using HEK293 cell monolayers. Three independent experiments performed with triplicates for each concentration. Baseline [Ca^2+^]_i_ ranged from 92 to113 nM. α-MSH-concentration-dependent activation of (B) hMC4R or (C) mMC4R (stably expressed in HEK293 cells) -induced [Ca^2+^]_i_ using cell suspensions. Three independent experiments performed with quadruplicates for each concentration. Baseline [Ca^2+^]_i_ ranged from 85 to 102 nM. Data shown as mean ± s.e.m.

Carbachol induces robust M3-AChR-activated mobilisation of [Ca^2+^]_i_ in HEK293 cell suspensions (Mountjoy *et al.* 2001). We used this carbachol response to validate the quantitative calcium assay on HEK293 cell monolayer. Three independent experiments produced carbachol-concentration-dependent curves for [Ca^2+^]_i_ with similar EC_50_ values (Fig. 8A). Pooling the data from these experiments determined the minimal (12.8 ± 4.4 nM), maximal (339.8 ± 3.9 nM), and EC_50_ (1.4 µM) carbachol-induced [Ca^2+^]_i_.

### The assay to quantitate carbachol-induced endogenous M3-AChR mobilisation of [Ca**^2+^**]_i_ in HEK293 cell monolayer lacks sensitivity to measure α-MSH-induced hMC4R or mMC4R mobilisation of [Ca**^2+^**]_i_

Previously, α-MSH produced concentration-dependent curves for stably expressed mMC4R-activated mobilisation of [Ca^2+^]_i_ in HEK293 cell suspensions (Mountjoy *et al.* 2001). Here, α-MSH was unable to stimulate an increase in [Ca^2+^]_i_ for hMC4R transiently transfected in HEK293 cell monolayers using the new calcium assay. Previously, the maximum α-MSH-activated mMC4R-induced [Ca^2+^]_i_ in HEK293 cell suspension was ~three-fold lower compared to the carbachol-activated M3-AChR-induced [Ca^2+^]_i_ (Mountjoy *et al.* 2001). We predicted that Pherastar FS might lack sensitivity to measure α-MSH-induced MC4R increase in [Ca^2+^]_i_ because the imaging aperture on PheraStar FS only views 1 mm of each well (each 96-plate well has a total area of ~ 0.36 cm^2^). Therefore, we modified the assay to increase the number of cells viewed by the PheraStar FS aperture.

### Validation of a new assay to quantitate α-MSH-induced hMC4R or mMC4R mobilisation of [Ca**^2+^**]_i_ in HEK293 cell suspensions in 96-well plates

We increased the number of cells viewed by the PheraStar FS aperture by loading cell suspensions into wells of a 96-well plate. We used stably expressed hMC4R or mMC4R in HEK293 cells, with Fura-2/AM loading and esterase activity taking place in cell suspensions. The buffers, incubation temperatures and incubation times used were those optimised for cell monolayers. The cells in suspension were then plated into a 96-well plate for imaging on PheraStar FS. The injection volume, injection speed, and volume of buffer in each well were optimised for use with cell suspensions in 96-well plates to eliminate stretch-receptor activation and maximise carbachol M3-AChR-induced [Ca^2+^]_i_. A 30 μL injection at 150 μL/s into a well containing 210 μL cell suspension consistently eliminated stretch-receptor-activated [Ca^2+^]_i_ response in HEK293 while providing a robust carbachol-induced increase for [Ca^2+^]_i_ (Supplementary Table 2). Using these optimised conditions, three independent experiments produced α-MSH-concentration-dependent curves for activation of hMC4R or mMC4R increasing [Ca^2+^]_i_, with similar EC_50_s for all experiments on each receptor (Fig. 8B). Data from the three independent experiments were pooled to determine the minimal (hMC4R: 24.1 ± 8.4 nM; mMC4R: 9.4 ± 4.1 nM), maximal (hMC4R: 206.1 ± 6.9 nM; mMC4R: 146.7 ± 2.5 nM), and EC_50_ (hMC4R: 1.4 nM; mMC4R: 0.4 nM) for α-MSH-activated MC4Rs-induced [Ca^2+^]_i_.

## Discussion

We developed a novel, high-throughput assay for quantitative measurements of ligand-activated MC4R-induced increases in [Ca^2+^]_i_. The assay conditions were optimised to eliminate interference from stretch-activated receptor increases in calcium and to maximise ligand-activated MC4R-induced [Ca^2+^]_i_. The latter was achieved using carbachol-activated endogenous M3-AChR-induced calcium signal as a positive control. The cell monolayer assay lacked sensitivity to detect increases in [Ca^2+^]_i_ following α-MSH-activated hMC4R transiently expressed in HEK293 cells. Therefore, a more sensitive assay was developed by plating cell suspensions into 96-well plates; this increased the number of cells monitored in each well compared with monolayers. The cell suspension assay was validated by showing reproducible α-MSH-concentration-dependent activation of stably expressed hMC4R or mMC4R in HEK293 cells inducing increases in [Ca^2+^]_i_, for three independent experiments. Stably expressing cells were used but we expect this method may also work for transiently transfected cells.

Interference from stretch-activated-receptor-induced [Ca^2+^]_i_ is a major problem when measuring ligand-activated GPCR-induced increases in [Ca^2+^]_i_ in cell monolayers (Demer *et al.* 1993, Naruse & Sokabe 1993, Sigurdson *et al.* 1993, Rosales *et al.* 1997, Tong *et al.* 1999, Hayakawa *et al.* 2008, Heusinkveld & Westerink 2011, Meijer *et al.* 2014). Activation of stretch receptor will flood the cell with calcium, and this has potential to either attenuate or synergise, subsequent ligand-activated-receptor-induced [Ca^2+^]_i_. Furthermore, activation of stretch-receptor-induced increase in [Ca^2+^]_i_ is highly variable between wells and assays; this likely contributes to current views that measurement of GPCR-induced mobilisation of [Ca^2+^]_i_ is not a reliable read-out in an overexpression system (Westerink & Hondebrink 2010, Meijer *et al.* 2014, Gillyard *et al.* 2019). We have developed an assay for high-throughput quantitative measurement of GPCR-induced mobilisation of [Ca^2+^]_i_ that is free of interference from stretch-activated-receptor-induced [Ca^2+^]_i_, by careful step-by-step optimisation of all assay conditions. Our data show that the stretch-activated-receptor-induced calcium response is sensitive to injection speed, injection volume, well-buffer volume, and well-buffer composition.

We discovered that while we could measure a robust carbachol-activated endogenous M3-AChR-induced [Ca^2+^]_i_ using cell monolayers, we could not detect α-MSH-activated transiently expressed hMC4R in cell monolayers inducing increased [Ca^2+^]_i_. High-throughput plate-reader based methods have a low-temporal and spatial resolution, resulting in low sensitivity for measuring highly dynamic and transient changes in [Ca^2+^]_i_ (Meijer *et al.* 2014) and we predicted that failure to detect an α-MSH/hMC4R response in cell monolayers was a sensitivity issue. Therefore, we developed an assay with an increased number of cells viewed by PheraStar FS 1 mm aperture in each well. For this assay, cells in suspension were loaded with Fura-2/AM, washed, and then incubated to enable esterase activity to release Fura-2, using the buffers, incubation temperatures and incubation times that had been optimised for use with cell monolayers. The cell suspension was plated into a 96-well plate to give 1.8 × 10^5^ cells/well, ready for imaging on the PheraStar FS. The estimated number of HEK293 cells in confluent monolayer in each well is ~1.2 × 10^5^ cells. Furthermore, the cells in suspension are more rounded compared to the adhered and flattened cells in monolayers; this potentially increases the number of plasma membrane-expressed hMC4R accessible to α-MSH activation, and also increases the number of cells viewed by the PheraStar FS aperture for cells in suspension, compared with monolayers. Following optimisation of injection volume and injection speed for cell suspensions, this assay had the sensitivity required to detect α-MSH activated hMC4R or mMC4R-induced [Ca^2+^]_i_, without interference from stretch-receptor-induced [Ca^2+^]_i_. Therefore, putting cell suspensions into 96-well plates for imaging on PheraStar FS increases the sensitivity for measuring highly dynamic and transient changes in [Ca^2+^]_i_ resulting from ligand-activation of GPCRs.

This quantitative assay to measure highly dynamic and transient changes in [Ca^2+^]_i_ will enable data to be compared between different assays, different laboratories, and different GPCRs. This contrasts with commercially available high-throughput assays for measuring transient changes in [Ca^2+^]_i_ that use single-wavelength calcium dyes, are not quantitative, and comparisons can only be made within assays. Commercially available assays frequently include probenecid in the dye-loading and esterase-activity buffers to retain the calcium dye inside cells. We show in the assay developed here that indeed probenecid enhances retention of calcium dye, but we also show that probenecid interferes with development of a quantitative assay for [Ca^2+^]_i_. EGTA is required to obtain the minimum [Ca^2+^]_i_, a measurement for the amount of Fura-2/AM loaded in each experiment. It is a standard procedure to inject EGTA onto cells to chelate calcium and thereby determine the Fura-2 fluorescence with minimal calcium present in the cell. This rapid EGTA chelation of calcium is visualised as a sudden marked decrease in fluorescence ratio (340/380). However, this does not occur in the presence of probenecid; addition of EGTA either has no effect on fluorescence ratio or increases the fluorescence ratio. While it is unknown why probenecid interferes with EGTA chelation of calcium, probenecid is known to produce a profound and prolonged attenuation of transmitter release when co-administered with calcium chelator, BAPTA/AM (Di Virgilio *et al.* 1988). To resolve the interference from probenecid, we plated cells into specific R_min_ wells for each assay, and [Ca^2+^]_i_ was depleted from the cells in these wells by the addition of EGTA prior to calcium imaging, when probenecid is required for Fura-2 retention. The esterase activity and calcium imaging buffer were also changed to a calcium-free buffer exclusively for these wells. All other conditions for these wells remained the same as the other wells in the assay. Following injection of EGTA into these ‘specific R_min_ wells’, there was consistently a rapid small decrease in fluorescence 340/380 ratio, indicative of EGTA chelation of calcium. We are not aware of other quantitative calcium assays using EGTA to deplete cells of calcium prior to calcium imaging.

We show good reproducibility for the assays developed here using either cell monolayers or cell suspensions in 96-well plates for quantitative measurement of [Ca^2+^]_i_. Three independent experiments for carbachol-activated M3-AChR in cell monolayers produced similar concentration-dependent curves, with almost identical EC_50_ values (Fig. 8A). Similarly, three independent experiments for α-MSH-activated hMC4R (Fig. 8B) or mMC4R (Fig. 8C) in cell suspensions produced comparable α-MSH concentration-dependent curves with similar EC_50_. Furthermore, data obtained in these high-throughput assays are not too different from data obtained in 2001 when a manual method was used to quantitate ligand-activated GPCR-induced [Ca^2+^]_i_ in HEK293 cell suspensions (Mountjoy *et al.* 2001). The manual method showed a peak minus baseline carbachol-activated M3-AChR-induced increase in [Ca^2+^]_i_ to ~290 nM with an EC_50_ = ~5 µM (Mountjoy *et al.* 2001), and here we observed a peak minus baseline carbachol-activated M3-AChR-induced increase in [Ca^2+^]_i_ equal to 340 nM (17% increase compared to manual method) with an EC_50_ = 1.4 µM (3.6% decrease compared to manual method). The manual method showed a peak minus baseline α-MSH-activated mMC4R-induced increase in [Ca^2+^]_i_ to ~100 nM with an EC_50_ = 0.5 nM (Mountjoy *et al.* 2001), and here we observed a peak minus baseline α-MSH-activated mMC4R-induced increase in [Ca^2+^]_i_ equal to 147 nM (11% increase compared to manual method) with an EC_50_ = 0.4 nM (1.3% decrease compared to manual method). The peak minus baseline α-MSH-activated hMC4R-induced increase in [Ca^2+^]_i_ equal to 206 nM with an EC_50_ = 1.4 nM. The higher but less sensitive calcium response observed here for hMC4R compared with mMC4R, reflects a potential species difference for MC4R coupling to [Ca^2+^]_i_ signalling pathway.

In summary, we have developed and validated a new high-throughput method to quantitate MC4R-induced mobilisation of [Ca^2+^]_i_ *in vitro*. This assay is reliable and reproducible from day-to-day and can therefore be used to compare quantitative transient changes in [Ca^2+^]_i_ for different ligands on MC4R or different GPCRs, with assays performed on different days, and in different laboratories. The high-throughput assay to quantitatively measure ligand-activated MC4R-induced [Ca^2+^]_i_ *in vitro* has potential to advance development of therapeutic drugs and understanding about MC4R signalling associated with human obesity. The utility of the assay in determining differences between Group V hMC4R variants and WT hMC4R remains to be determined.

## Supplementary Material

Table S1: Results for optimisation of injection volume and injection speed on HEK293 cell monolayers to eliminate vehicle-induced stretch-activated calcium response.

Table S2: Results for optimisation of injection volume and injection speed on HEK293 cell suspensions to eliminate vehicle-induced stretch-activated calcium response.

Figure 1: Schematic showing the experimental parameters optimised for measuring [Ca2+]i in HEK293 cell monolayers.

Figure 2: Representative trace of fluorescence kinetic data from single-wells showing stretch-receptor, vehicle and agonist-induced [Ca2+]i in HEK293 cell monolayers.

Figure 3: Optimal buffer for Fura-2/AM loading is DMEM and optimal buffer for esterase cleavage and calcium imaging is CLB-HG.

Figure 4: Optimal temperature for Fura-2/AM loading is RT and the optimal temperature for esterase cleavage of Fura-2/AM is 37°C.

Figure 5: Optimal incubation time for Fura-2/AM loading is 60 min and optimal incubation time for cytosolic esterase cleavage of Fura-2/AM is 80 min.

Figure 6: Pluronic acid and probenecid enhanced carbachol-activated M3-AChR-induced calcium signal.

Figure 7: EGTA sequestration of calcium during fura-2/AM loading enabled use of EGTA injection to chelate calcium for determination of Rmin for quantitation [Ca2+]i.

Figure 8: Validation of high-throughput quantitative ligand-activated GPCR-induced [Ca2+]i assay using cell monolayers or cell suspensions.

## Declaration of interest

The authors declare that there is no conflict of interest that could be perceived as prejudicing the impartiality of the research reported.

## Funding

Funding was provided from The University of Auckland postgraduate student fund.

## Author contribution statement

S S K and K G M conceptualisation ; S S K data curation and formal analysis; M-L W and K G M methodology; K G M supervision; S S K writing original draft; M-L W and K G M editing.

## References

[bib1] Chai B Li JY Zhang W Newman E Ammori JB Mulholland MW 2006 Melanocortin-4 receptor-mediated inhibition of apoptosis in immortalized hypothalamic neurons via mitogen-activated protein kinase. Peptides 27 2846–2857. (10.1016/j.peptides.2006.05.005) 16806584

[bib2] Clement K Biebermann H Farooqi IS Van Der Ploeg L Wolters B Poitou C Puder L Fiedorek F Gottesdiener K Kleinau G***et al***. 2018 MC4R agonism promotes durable weight loss in patients with leptin receptor deficiency. Nature Medicine 24 551–555. (10.1038/s41591-018-0015-9) 29736023

[bib3] Daniels D Patten CS Roth JD Yee DK Fluharty SJ 2003 Melanocortin receptor signaling through mitogen-activated protein kinase in vitro and in rat hypothalamus. Brain Research 986 1–11. (10.1016/s0006-8993(0303162-7) 12965224

[bib4] Demer LL Wortham CM Dirksen ER Sanderson MJ 1993 Mechanical stimulation induces intercellular calcium signaling in bovine aortic endothelial cells. American Journal of Physiology 264 H2094–H2102. (10.1152/ajpheart.1993.264.6.H2094) 8322938

[bib5] Di Virgilio F Steinberg TH Swanson JA Silverstein SC 1988 Fura-2 secretion and sequestration in macrophages. A blocker of organic anion transport reveals that these processes occur via a membrane transport system for organic anions. Journal of Immunology 140 915–920. 3339244

[bib6] Di Virgilio F Steinberg TH Silverstein SC 1990 Inhibition of Fura-2 sequestration and secretion with organic anion transport blockers. Cell Calcium 11 57–62. (10.1016/0143-4160(9090059-4) 2191781

[bib7] Farooqi IS Yeo GS Keogh JM Aminian S Jebb SA Butler G Cheetham T O’Rahilly S 2000 Dominant and recessive inheritance of morbid obesity associated with melanocortin 4 receptor deficiency. Journal of Clinical Investigation 106 271–279. (10.1172/JCI9397) PMC314308 10903343

[bib8] Galaz-Montoya M Wright SJ Rodriguez GJ Lichtarge O Wensel TG 2017 Beta2-adrenergic receptor activation mobilizes intracellular calcium via a non-canonical cAMP-independent signaling pathway. Journal of Biological Chemistry 292 9967–9974. (10.1074/jbc.M117.787119) PMC5473248 28442571

[bib9] Gillyard T Fowler K Williams SY Cone RD 2019 Obesity-associated mutant melanocortin-4 receptors with normal Galphas coupling frequently exhibit other discoverable pharmacological and biochemical defects. Journal of Neuroendocrinology 31 e12795. (10.1111/jne.12795) 31529534

[bib10] Grynkiewicz G Poenie M Tsien RY 1985 A new generation of Ca2+ indicators with greatly improved fluorescent properties. Journal of Biological Chemistry 260 3440–3450. (10.1016/S0021-9258(1983641-4) 3838314

[bib11] Hayakawa K Tatsumi H Sokabe M 2008 Actin stress fibers transmit and focus force to activate mechanosensitive channels. Journal of Cell Science 121 496–503. (10.1242/jcs.022053) 18230647

[bib12] Heusinkveld HJ Westerink RH 2011 Caveats and limitations of plate reader-based high-throughput kinetic measurements of intracellular calcium levels. Toxicology and Applied Pharmacology 255 1–8. (10.1016/j.taap.2011.05.020) 21684299

[bib13] Hoth M Penner R 1992 Depletion of intracellular calcium stores activates a calcium current in mast cells. Nature 355 353–356. (10.1038/355353a0) 1309940

[bib14] Kay EI Botha R Montgomery JM Mountjoy KG 2015 hMRAPalpha, but not hMRAP2, enhances hMC4R constitutive activity in HEK293 cells and this is not dependent on hMRAPalpha induced changes in hMC4R complex N-linked glycosylation. PLoS ONE 10 e0140320. (10.1371/journal.pone.0140320) PMC4607451 26469516

[bib15] Krylova OO Pohl P 2004 Ionophoric activity of pluronic block copolymers. Biochemistry 43 3696–3703. (10.1021/bi035768l) 15035640

[bib16] Kurko D Bekes Z Gere A Baki A Boros A Kolok S Bugovics G Nagy J Szombathelyi Z Ignacz-Szendrei G 2009 Comparative pharmacology of adrenergic alpha(2C) receptors coupled to Ca(2+) signaling through different Galpha proteins. Neurochemistry International 55 467–475. (10.1016/j.neuint.2009.04.015) 19426776

[bib17] Li X Llorente I Brasch M 2008 Improvements in live cell analysis of G protein coupled receptors using second generation BD calcium assay kits. Current Chemical Genomics 2 10–15. (10.2174/1875397300802010010) 20161839 PMC2803433

[bib18] Lotta LA Mokrosinski J Mendes De Oliveira E Li C Sharp SJ Luan J Brouwers B Ayinampudi V Bowker N Kerrison N***et al***. 2019 Human gain-of-function MC4R variants show signaling bias and protect against obesity. Cell 177 597.e9–607.e9. (10.1016/j.cell.2019.03.044) PMC6476272 31002796

[bib19] Malgaroli A Milani D Meldolesi J Pozzan T 1987 Fura-2 measurement of cytosolic free Ca2+ in monolayers and suspensions of various types of animal cells. Journal of Cell Biology 105 2145–2155. (10.1083/jcb.105.5.2145) PMC2114834 3680375

[bib20] Meijer M Hendriks HS Heusinkveld HJ Langeveld WT Westerink RH 2014 Comparison of plate reader-based methods with fluorescence microscopy for measurements of intracellular calcium levels for the assessment of in vitro neurotoxicity. Neurotoxicology 45 31–37. (10.1016/j.neuro.2014.09.001) 25224521

[bib21] Mountjoy KG Mortrud MT Low MJ Simerly RB Cone RD 1994 Localization of the melanocortin-4 receptor (MC4-R) in neuroendocrine and autonomic control circuits in the brain. Molecular Endocrinology 8 1298–1308. (10.1210/mend.8.10.7854347) 7854347

[bib22] Mountjoy KG Kong PL Taylor JA Willard DH Wilkison WO 2001 Melanocortin receptor-mediated mobilization of intracellular free calcium in HEK293 cells. Physiological Genomics 5 11–19. (10.1152/physiolgenomics.2001.5.1.11) 11161002

[bib23] Naruse K Sokabe M 1993 Involvement of stretch-activated ion channels in Ca2+ mobilization to mechanical stretch in endothelial cells. American Journal of Physiology 264 C1037–C1044. (10.1152/ajpcell.1993.264.4.C1037) 8386448

[bib24] Newman EA Chai BX Zhang W Li JY Ammori JB Mulholland MW 2006 Activation of the melanocortin-4 receptor mobilizes intracellular free calcium in immortalized hypothalamic neurons. Journal of Surgical Research 132 201–207. (10.1016/j.jss.2006.02.003) 16580690

[bib25] O’Connor N Silver RB 2013 Ratio imaging: practical considerations for measuring intracellular Ca2+ and pH in living cells. Methods in Cell Biology 114 387–406. (10.1016/B978-0-12-407761-4.00016-6) 23931515

[bib26] Paisdzior S Dimitriou IM Schope PC Annibale P Scheerer P Krude H Lohse MJ Biebermann H Kuhnen P 2020 Differential signaling profiles of MC4R mutations with three different ligands. International Journal of Molecular Sciences 21 1224. (10.3390/ijms21041224) PMC7072973 32059383

[bib27] Paredes RM Etzler JC Watts LT Zheng W Lechleiter JD 2008 Chemical calcium indicators. Methods 46 143–151. (10.1016/j.ymeth.2008.09.025) 18929663 PMC2666335

[bib28] Roe MW Lemasters JJ Herman B 1990 Assessment of Fura-2 for measurements of cytosolic free calcium. Cell Calcium 11 63–73. (10.1016/0143-4160(9090060-8) 2191782

[bib29] Rosales OR Isales CM Barrett PQ Brophy C Sumpio BE 1997 Exposure of endothelial cells to cyclic strain induces elevations of cytosolic Ca2+ concentration through mobilization of intracellular and extracellular pools. Biochemical Journal 326 385–392. (10.1042/bj3260385) PMC1218682 9291109

[bib30] Sauve R Diarra A Chahine M Simoneau C Garneau L Roy G 1990 Single-channel and Fura-2 analysis of internal Ca2+ oscillations in HeLa cells: contribution of the receptor-evoked Ca2+ influx and effect of internal pH. Pflugers Archiv 416 43–52. (10.1007/BF00370220) 2352841

[bib31] Sharma S Thibodeau S Lytton J 2020 Signal pathway analysis of selected obesity-associated melanocortin-4 receptor class V mutants. Biochimica et Biophysica Acta: Molecular Basis of Disease 1866 165835. (10.1016/j.bbadis.2020.165835) 32423884

[bib32] Sigurdson WJ Sachs F Diamond SL 1993 Mechanical perturbation of cultured human endothelial cells causes rapid increases of intracellular calcium. American Journal of Physiology 264 H1745–H1752. (10.1152/ajpheart.1993.264.6.H1745) 8322903

[bib33] Struk A Szucs G Kemmer H Melzer W 1998 Fura-2 calcium signals in skeletal muscle fibres loaded with high concentrations of EGTA. Cell Calcium 23 23–32. (10.1016/s0143-4160(9890071-9) 9570007

[bib34] Tong J Du GG Chen SR Maclennan DH 1999 HEK-293 cells possess a carbachol- and thapsigargin-sensitive intracellular Ca2+ store that is responsive to stop-flow medium changes and insensitive to caffeine and ryanodine. Biochemical Journal 343 39–44. PMC1220521 10493909

[bib35] Vaisse C Clement K Durand E Hercberg S Guy-Grand B Froguel P 2000 Melanocortin-4 receptor mutations are a frequent and heterogeneous cause of morbid obesity. Journal of Clinical Investigation 106 253–262. (10.1172/JCI9238) PMC314306 10903341

[bib36] Valentine WJ Tigyi G 2012 High-throughput assays to measure intracellular Ca(2)(+) mobilization in cells that express recombinant S1P receptor subtypes. Methods in Molecular Biology 874 77–87. (10.1007/978-1-61779-800-9_7) 22528441 PMC3617928

[bib37] Vongs A Lynn NM Rosenblum CI 2004 Activation of MAP kinase by MC4-R through PI3 kinase. Regulatory Peptides 120 113–118. (10.1016/j.regpep.2004.02.018) 15177928

[bib38] Ward ML Pope AJ Loiselle DS Cannell MB 2003 Reduced contraction strength with increased intracellular [Ca2+] in left ventricular trabeculae from failing rat hearts. Journal of Physiology 546 537–550. (10.1113/jphysiol.2002.029132) PMC2342526 12527740

[bib39] Westerink RH Hondebrink L 2010 Are high-throughput measurements of intracellular calcium using plate-readers sufficiently accurate and reliable? Toxicology and Applied Pharmacology 249 247–248; author reply 249–250. (10.1016/j.taap.2010.09.014) 20869982

[bib40] Wright PT Schobesberger S Gorelik J 2015 Studying GPCR/cAMP pharmacology from the perspective of cellular structure. Frontiers in Pharmacology 6 148. (10.3389/fphar.2015.00148) PMC4505077 26236239

[bib41] Yeo GS Lank EJ Farooqi IS Keogh J Challis BG O’Rahilly S 2003 Mutations in the human melanocortin-4 receptor gene associated with severe familial obesity disrupts receptor function through multiple molecular mechanisms. Human Molecular Genetics 12 561–574. (10.1093/hmg/ddg057) 12588803

[bib42] Zhang L Cannell MB Phillips AR Cooper GJ Ward ML 2008 Altered calcium homeostasis does not explain the contractile deficit of diabetic cardiomyopathy. Diabetes 57 2158–2166. (10.2337/db08-0140) 18492789 PMC2494698

