# Supplementary material for: Quantitative high-throughput assay to measure MC4R-induced intracellular calcium
Source: J Mol Endocrinol. 2021 Mar 19;66(4):285–97. doi: 10.1530/JME-20-0285 (PMC8111326; doi:10.1530/JME-20-0285)
Supplement: Table S1: Results for optimisation of injection volume and injection speed on HEK293 cell monolayers to eliminate vehicle-induced stretch-activated calcium response. [file supplementary_table_1.pdf]

Table S1: Results for optimisation of injection volume and injection speed on HEK293 cell monolayers to eliminate vehicle-induced stretch-activated calcium response.

		Injection speed (µL/s)																																			
		100			115			135			150			170			190			220			260			300			350			400			430		
		Replicates																																			
**Injection volume (µL)**	**Experiment**	1	2	3	1	2	3	1	2	3	1	2	3	1	2	3	1	2	3	1	2	3	1	2	3	1	2	3	1	2	3	1	2	3	1	2	3
**10**	1	-	-	-	-	-	-	-	+	-	+	+	+	+	-	+	+	+	+	-	+	+	+	+	+	+	+	+	+	+	+	+	+	+	+	+	+
	2	-	-	-	-	+	-	-	-	+	-	-	-	-	-	-	-	-	-	-	-	-	-	-	-	-	-	-	+	+	+	+	+	+	+	+	+
	3	-	-	-	-	-	-	-	-	-	-	-	+	-	-	-	-	-	-	+	-	-	-	-	-	+	+	-	-	-	+	+	+	+	+	+	+
**20**	1	-	-	-	-	+	+	-	+	+	+	+	+	n	n	n	n	n	n	n	n	n	n	n	n	n	n	n	n	n	n	n	n	n	n	n	n
	2	-	-	-	+	+	+	+	+	+	+	-	+	n	n	n	n	n	n	n	n	n	n	n	n	n	n	n	n	n	n	n	n	n	n	n	n
	3	+	-	-	+	-	+	+	-	-	+	+	+	n	n	n	n	n	n	n	n	n	n	n	n	n	n	n	n	n	n	n	n	n	n	n	n
**30**	1	-	+	-	+	-	+	+	+	+	n	n	n	n	n	n	n	n	n	n	n	n	n	n	n	n	n	n	n	n	n	n	n	n	n	n	n
	2	-	-	-	-	-	-	+	-	-	n	n	n	n	n	n	n	n	n	n	n	n	n	n	n	n	n	n	n	n	n	n	n	n	n	n	n
	3	-	-	-	-	-	+	+	+	-	n	n	n	n	n	n	n	n	n	n	n	n	n	n	n	n	n	n	n	n	n	n	n	n	n	n	n
**40**	1	-	-	-	-	-	+	n	n	n	n	n	n	n	n	n	n	n	n	n	n	n	n	n	n	n	n	n	n	n	n	n	n	n	n	n	n
	2	+	+	+	+	+	+	n	n	n	n	n	n	n	n	n	n	n	n	n	n	n	n	n	n	n	n	n	n	n	n	n	n	n	n	n	n
	3	-	-	+	+	-	+	n	n	n	n	n	n	n	n	n	n	n	n	n	n	n	n	n	n	n	n	n	n	n	n	n	n	n	n	n	n
**50**	1	+	+	+	n	n	n	n	n	n	n	n	n	n	n	n	n	n	n	n	n	n	n	n	n	n	n	n	n	n	n	n	n	n	n	n	n
	2	+	-	+	n	n	n	n	n	n	n	n	n	n	n	n	n	n	n	n	n	n	n	n	n	n	n	n	n	n	n	n	n	n	n	n	n
	3	+	+	+	n	n	n	n	n	n	n	n	n	n	n	n	n	n	n	n	n	n	n	n	n	n	n	n	n	n	n	n	n	n	n	n	n

(+) = activation of stretch receptor; (-) = no activation of stretch receptor; (n) = no data available.

Data collated from three independent experiments with each condition tested in triplicate (n=9).

Green shading = The optimal injection speed and injection volume chosen for use with cell monolayers. Based on these data, a 10 µL injection volume injected at 100 µL/s onto 290 µL buffer covering the cell monolayer prevented stretch-activated calcium response. The final buffer volume in each well was always maintained at 300 µL.
