# Supplementary material for: Quantitative high-throughput assay to measure MC4R-induced intracellular calcium
Source: J Mol Endocrinol. 2021 Mar 19;66(4):285–97. doi: 10.1530/JME-20-0285 (PMC8111326; doi:10.1530/JME-20-0285)
Supplement: Table S2: Results for optimisation of injection volume and injection speed on HEK293 cell suspensions to eliminate vehicle-induced stretch-activated calcium response. [file supplementary_table_2.pdf]

Table S2: Results for optimisation of injection volume and injection speed on HEK293 cell suspensions to eliminate vehicle-induced stretch-activated calcium response.

	Injection speed (µL/s)	100									115									130									150								
	Injection volume (µL)	10			20			30			10			20			30			10			20			30			10			20			30		
		Replicates																																			
Final Well volume (µL)	Experiment	1	2	3	1	2	3	1	2	3	1	2	3	1	2	3	1	2	3	1	2	3	1	2	3	1	2	3	1	2	3	1	2	3	1	2	3
290	1	-	+	-	-	-	-	-	+	-	-	-	-	+	-	-	-	-	-	-	-	+	-	-	-	-	-	-	+	-	-	-	+	-	-	-	-
	2	-	-	-	-	-	-	-	-	-	-	-	-	-	-	-	-	-	-	-	-	-	-	-	+	-	-	-	-	-	-	-	-	-	-	-	-
	3	-	-	-	-	-	-	-	-	+	-	-	-	-	-	-	-	-	-	-	-	-	-	-	-	-	-	-	-	-	-	-	-	-	-	-	-
280	1	-	-	-	-	+	-	-	-	-	-	-	-	-	-	+	-	-	-	-	-	-	-	-	-	-	-	-	-	-	-	-	-	+	-	-	-
	2	-	-	-	-	-	-	-	-	-	-	-	-	-	-	-	-	-	-	-	-	-	-	+	-	-	-	-	-	-	-	-	-	-	-	+	-
	3	+	-	-	-	-	-	-	-	-	-	-	-	-	-	-	-	-	-	-	-	-	-	-	-	-	-	-	-	-	-	-	-	-	-	-	-
270	1	-	-	-	+	-	-	-	-	-	-	-	+	-	-	-	-	-	-	-	-	-	-	-	-	-	-	-	-	-	+	-	-	-	-	-	-
	2	-	-	-	-	-	-	-	-	-	+	-	-	-	-	-	-	-	-	-	-	-	-	-	-	-	-	-	+	-	-	-	-	-	-	-	+
	3	-	-	-	-	-	-	-	-	-	-	-	-	-	-	+	-	-	-	-	-	-	-	-	-	-	-	-	-	-	-	-	-	-	-	-	-
260	1	-	-	-	-	-	+	-	-	-	-	-	-	-	-	-	-	-	-	-	-	-	-	-	-	-	-	-	-	-	-	-	-	-	-	+	-
	2	-	-	-	-	-	-	-	-	-	-	-	-	-	+	-	-	-	-	-	+	-	-	-	-	-	-	-	+	-	-	-	-	-	-	-	-
	3	-	-	+	-	-	-	-	-	-	-	-	-	-	-	-	-	-	-	-	-	-	-	-	-	-	+	-	-	-	+	-	-	-	-	-	-
250	1	+	-	-	-	-	-	-	-	-	-	-	-	-	-	-	-	-	-	-	-	-	-	-	-	-	-	-	-	-	-	-	-	-	-	+	-
	2	-	-	-	-	-	-	-	-	-	-	-	-	-	-	-	-	-	-	-	+	-	-	-	-	-	-	-	-	-	-	-	+	-	-	-	-

	3	-	-	-	-	-	-	-	-	-	-	-	-	-	-	-	-	-	-	-	-	-	-	-	-	-	+	-	-	-	-	-	-	-	-	-	-
**240**	1	-	-	-	-	-	-	-	+	-	-	-	-	-	-	-	-	-	-	-	+	-	-	-	-	-	-	-	-	-	-	-	+	-	-	-	-
	2	-	-	-	-	-	-	-	-	-	-	-	-	-	-	-	-	-	-	-	-	-	-	-	-	-	-	-	-	-	-	-	-	-	-	-	-
	3	-	-	-	-	-	-	-	-	-	-	-	-	-	-	-	-	-	-	-	-	-	-	-	-	-	-	-	-	-	-	-	-	-	-	-	-
**230**	1	-	-	-	-	-	+	+	-	-	-	-	-	-	-	-	-	-	+	-	-	-	-	+	-	-	+	-	-	-	-	-	+	+	-	+	-
	2	+	-	-	-	+	+	-	-	-	-	+	-	-	-	+	-	-	+	-	-	+	+	-	+	-	-	+	+	-	-	+	-	-	-	-	-
	3	-	-	-	+	+	+	-	-	-	+	-	+	+	+	+	+	-	-	-	+	+	+	-	-	-	+	-	+	+	+	+	+	-	-	-	-
**220**	1	+	-	-	+	-	+	-	-	-	+	+	+	+	-	-	-	+	-	-	+	-	+	-	-	-	+	+	+	+	-	-	-	+	-	-	+
	2	-	+	-	+	-	+	+	-	-	-	-	+	-	-	-	+	-	+	-	+	-	+	+	+	+	+	+	+	-	-	-	+	+	+	+	+
	3	-	+	+	+	+	+	-	-	-	+	-	-	+	+	+	+	-	-	+	+	+	+	+	-	-	+	-	-	+	+	+	+	+	+	-	-
**210**	1	-	-	-	-	-	+	-	-	-	+	+	-	-	-	+	+	-	+	+	+	+	+	-	+	-	+	+	-	-	-	+	+	+	-	-	-
	2	-	-	-	+	+	+	-	+	-	+	-	+	+	+	+	+	-	-	-	+	+	+	-	-	-	+	-	+	+	+	+	+	+	+	+	+
	3	+	-	-	+	-	+	-	-	-	+	+	+	+	-	-	-	+	-	-	+	-	+	-	+	-	+	+	+	+	+	+	+	+	+	-	+

(+) = activation of stretch receptor; (-) = no activation of stretch receptor.

Data collated from three independent experiments with each condition tested in triplicate (n=9).

Blue shading = The optimal injection speed and injection volume chosen for use with cell suspensions. Based on these data, a 30 µL injection volume injected at 150 µL/s onto 210 µL buffer with cells in suspension prevented stretch-activated calcium response.
