# Supplementary material for: Quantitative high-throughput assay to measure MC4R-induced intracellular calcium
Source: J Mol Endocrinol. 2021 Mar 19;66(4):285–97. doi: 10.1530/JME-20-0285 (PMC8111326; doi:10.1530/JME-20-0285)
Supplement: Figure 1: Schematic showing the experimental parameters optimised for measuring [Ca2+]i in HEK293 cell monolayers. [file supplementary_figure_1.pdf]

Figure S1

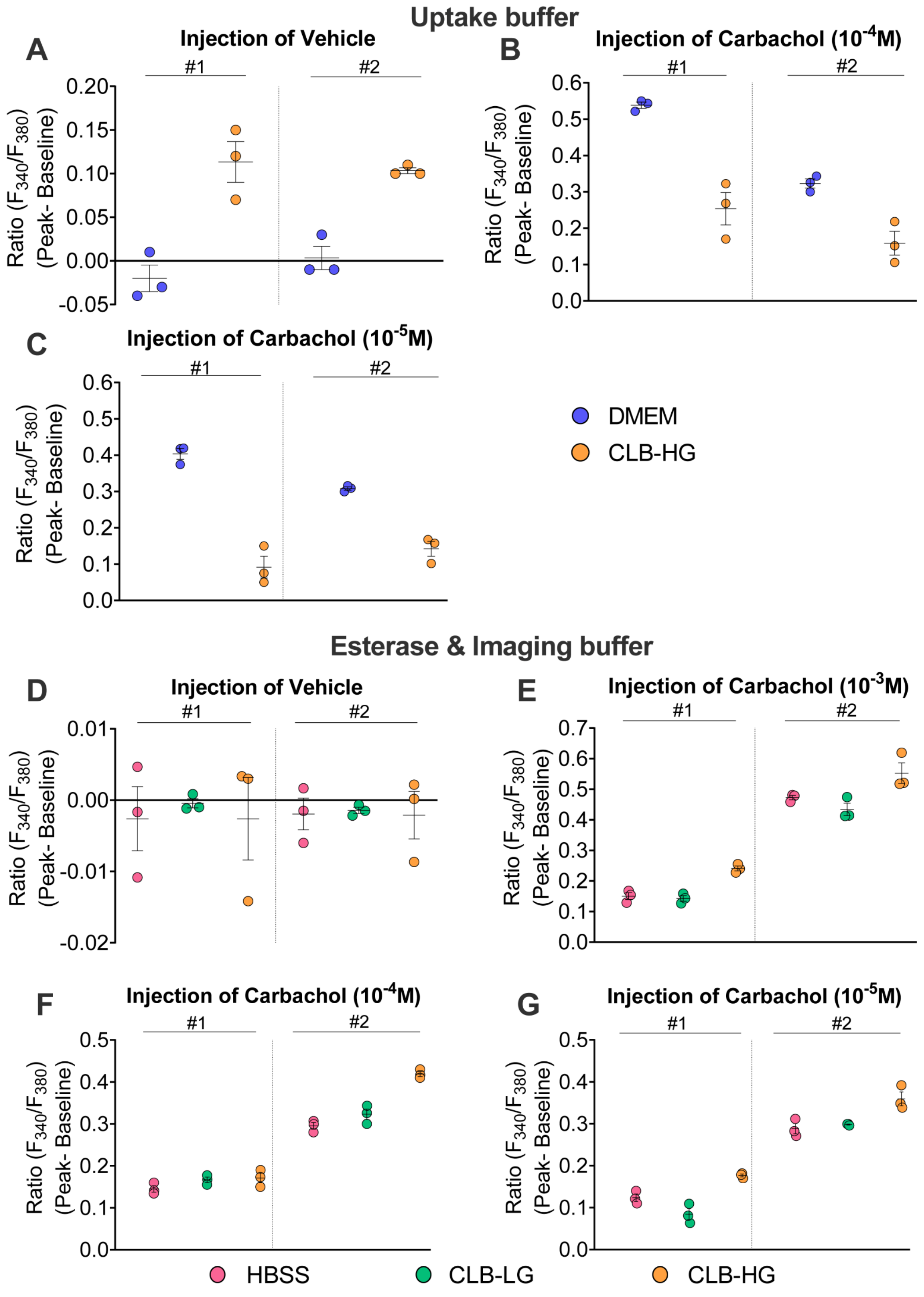

Figure S1: Optimal buffer for Fura-2/AM loading is DMEM, and optimal buffer for esterase cleavage and calcium imaging is CLB-HG. Cells were tested for vehicle-induced stretch-activated calcium response (A & D) and carbachol (B, C & E-G) induced calcium signal. Data shown as mean ± S.E.M for two independent experiments with three replicates in each experiment.
