# Supplementary material for: Quantitative high-throughput assay to measure MC4R-induced intracellular calcium
Source: J Mol Endocrinol. 2021 Mar 19;66(4):285–97. doi: 10.1530/JME-20-0285 (PMC8111326; doi:10.1530/JME-20-0285)
Supplement: Figure 2: Representative trace of fluorescence kinetic data from single-wells showing stretch-receptor, vehicle and agonist-induced [Ca2+]i in HEK293 cell monolayers. [file supplementary_figure_2.pdf]

Figure S2

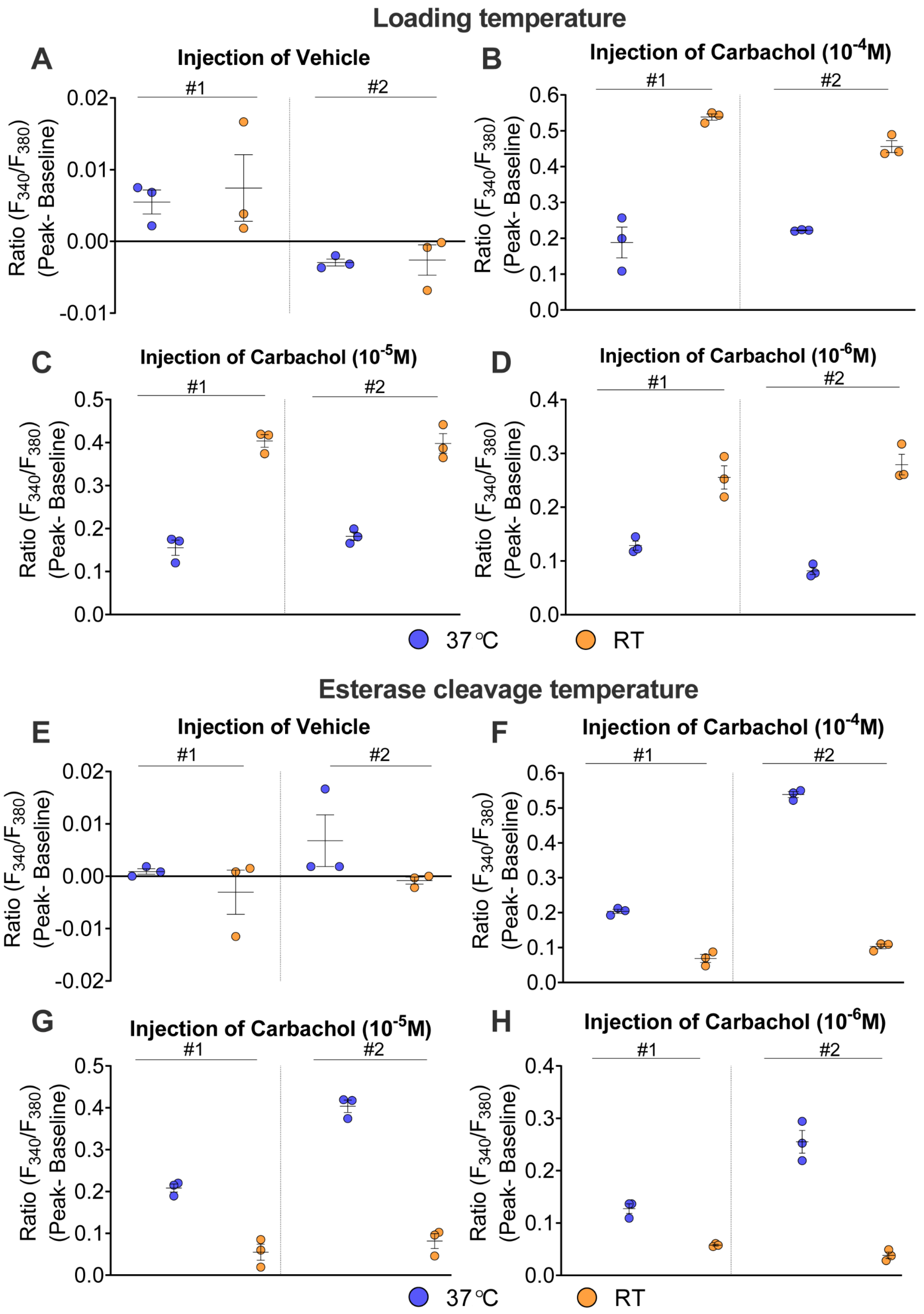

Figure S2: Optimal temperature for Fura-2/AM loading is room temperature, and the optimal temperature for esterase cleavage of Fura-2/AM is 37°C. Cells were tested for vehicle-induced stretch-activated calcium response (A & E) and carbachol (B-D & F-H) induced calcium signal. Data shown as mean ± S.E.M for two independent experiments with three replicates in each experiment.
