# Supplementary material for: Quantitative high-throughput assay to measure MC4R-induced intracellular calcium
Source: J Mol Endocrinol. 2021 Mar 19;66(4):285–97. doi: 10.1530/JME-20-0285 (PMC8111326; doi:10.1530/JME-20-0285)
Supplement: Figure 4: Optimal temperature for Fura-2/AM loading is RT and the optimal temperature for esterase cleavage of Fura-2/AM is 37°C. [file supplementary_figure_4.pdf]

Figure S4

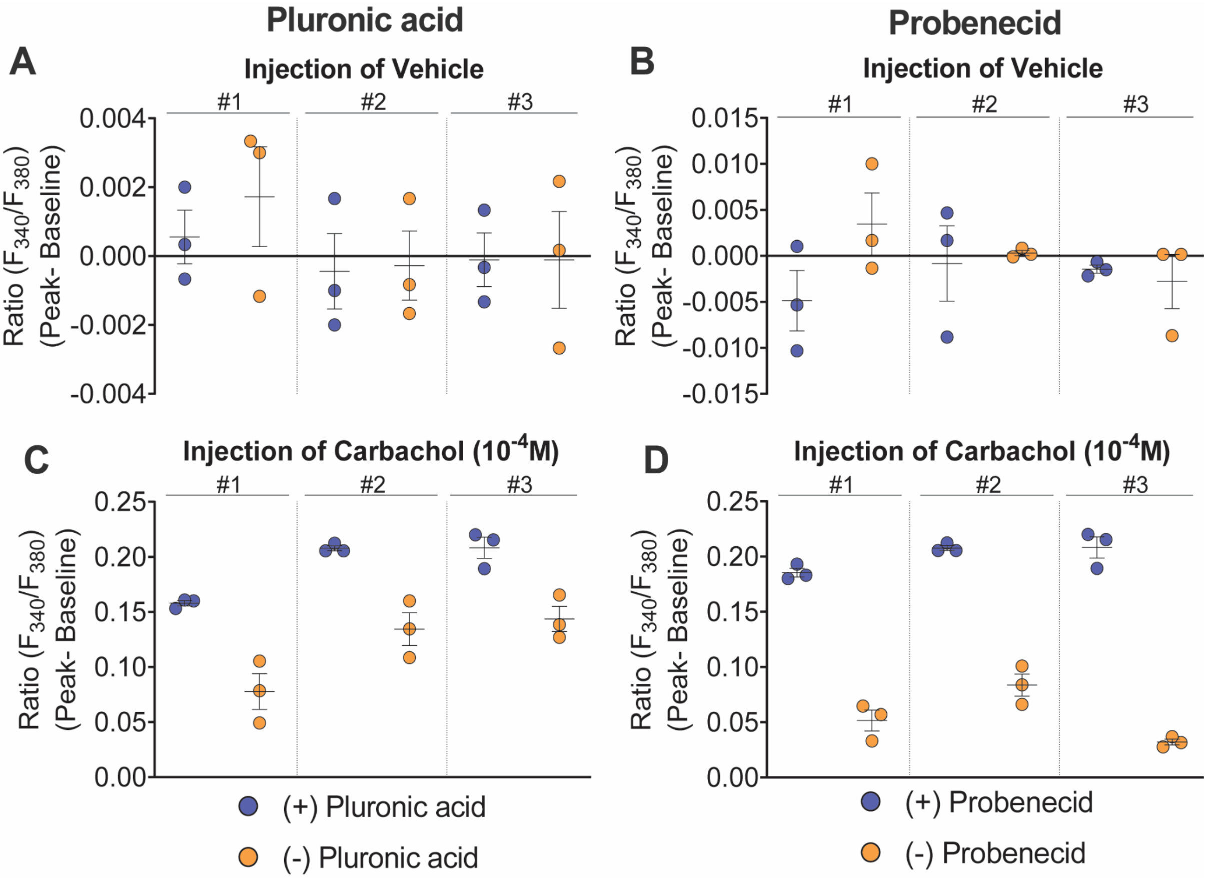

Figure S4: Addition of pluronic acid to loading buffer and probenecid to both loading and esterase cleavage buffers is needed for optimal Fura-2/AM loading and calcium imaging. Cells were tested for vehicle-induced stretch-activated calcium response (A & B) and carbachol (C & D) induced calcium signal. Data shown as mean ± S.E.M for three independent experiments with three replicates in each experiment.
