# Supplementary material for: Quantitative high-throughput assay to measure MC4R-induced intracellular calcium
Source: J Mol Endocrinol. 2021 Mar 19;66(4):285–97. doi: 10.1530/JME-20-0285 (PMC8111326; doi:10.1530/JME-20-0285)
Supplement: Figure 5: Optimal incubation time for Fura-2/AM loading is 60 min and optimal incubation time for cytosolic esterase cleavage of Fura-2/AM is 80 min. [file supplementary_figure_5.pdf]

Figure S5

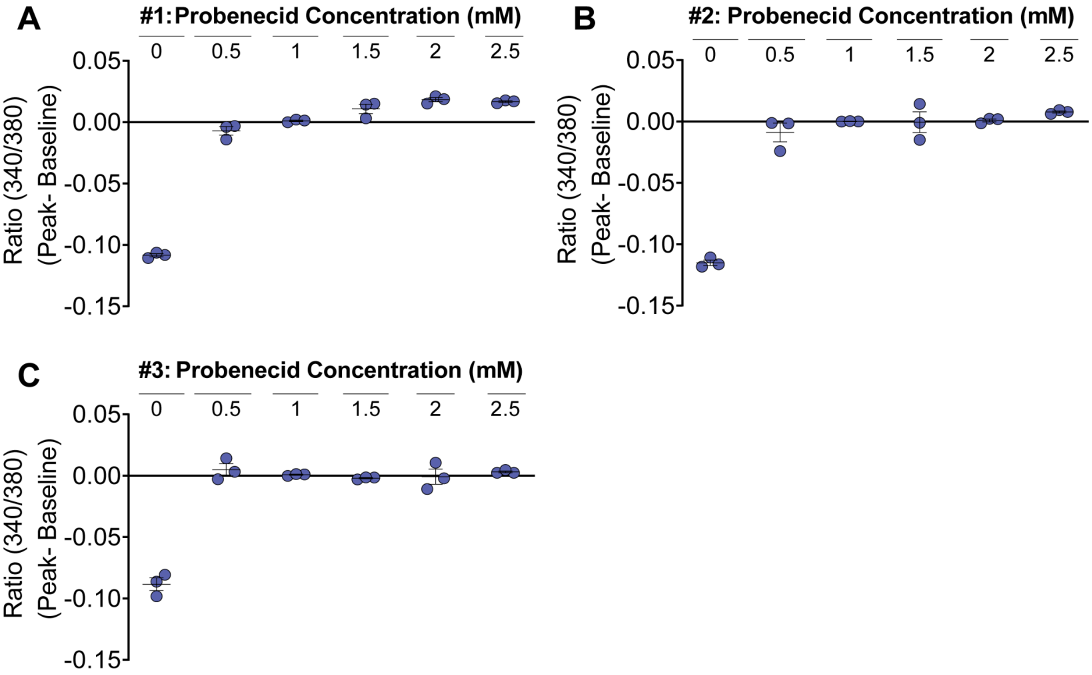

Figure S5: EGTA chelation of calcium tested with various concentrations of probenecid. Cells loaded with Fura-2/AM in the absence or presence of probenecid (0.5 - 2.5 mM) were stimulated with EGTA (A-C). Data shown as mean ± S.E.M for three independent experiments with three replicates in each experiment.
