# Supplementary material for: Quantitative high-throughput assay to measure MC4R-induced intracellular calcium
Source: J Mol Endocrinol. 2021 Mar 19;66(4):285–97. doi: 10.1530/JME-20-0285 (PMC8111326; doi:10.1530/JME-20-0285)
Supplement: Figure 6: Pluronic acid and probenecid enhanced carbachol-activated M3-AChR-induced calcium signal. [file supplementary_figure_6.pdf]

Figure S6

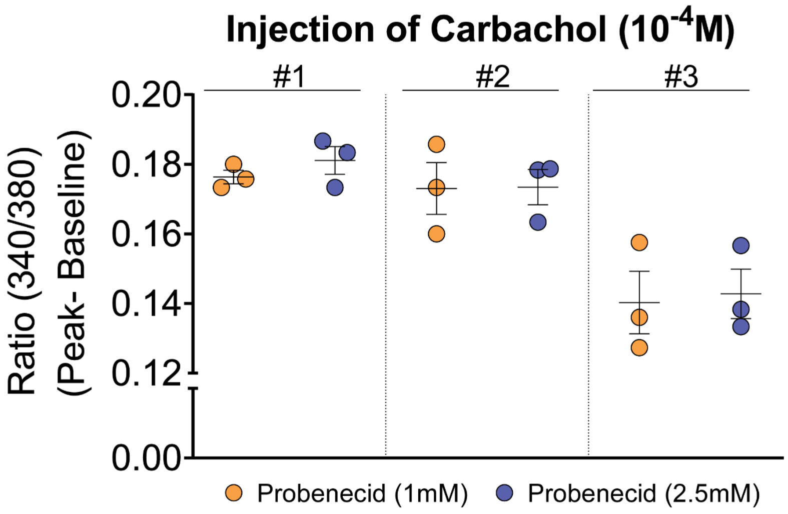

Figure S6: Carbachol-stimulated calcium response in the presence of different concentrations of probenecid. Fura-2/AM loaded cells were stimulated with carbachol 10^{-4}M following the addition of probenecid (1 mM or 2.5 mM) to the esterase cleavage buffer. Data shown as mean ± S.E.M for three independent experiments with three replicates in each experiment.
