# Supplementary material for: Quantitative high-throughput assay to measure MC4R-induced intracellular calcium
Source: J Mol Endocrinol. 2021 Mar 19;66(4):285–97. doi: 10.1530/JME-20-0285 (PMC8111326; doi:10.1530/JME-20-0285)
Supplement: Figure 7: EGTA sequestration of calcium during fura-2/AM loading enabled use of EGTA injection to chelate calcium for determination of Rmin for quantitation [Ca2+]i. [file supplementary_figure_7.pdf]

Figure S7

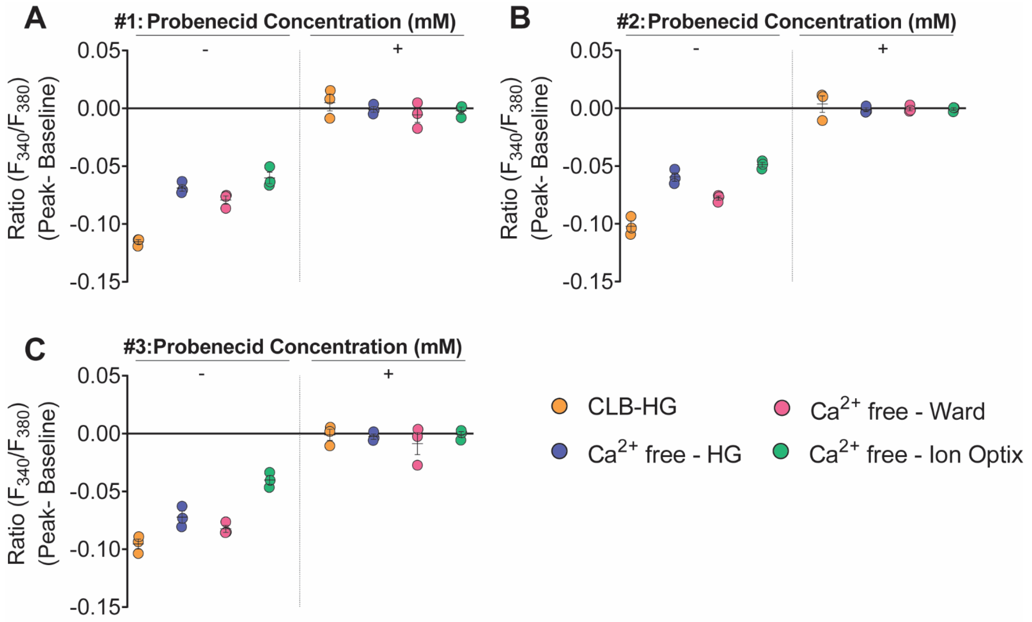

Figure S7: EGTA chelation of calcium tested with different calcium buffers in the absence or presence of probenecid. Cells loaded with Fura-2/AM in the absence or presence of probenecid (1mM) were stimulated with EGTA in calcium containing and calcium-free buffers (A-C). Data shown as mean ± S.E.M for three independent experiments with three replicates in each experiment.
