# Supplementary material for: Quantitative high-throughput assay to measure MC4R-induced intracellular calcium
Source: J Mol Endocrinol. 2021 Mar 19;66(4):285–97. doi: 10.1530/JME-20-0285 (PMC8111326; doi:10.1530/JME-20-0285)
Supplement: Figure 8: Validation of high-throughput quantitative ligand-activated GPCR-induced [Ca2+]i assay using cell monolayers or cell suspensions. [file supplementary_figure_8.pdf]

Figure S8

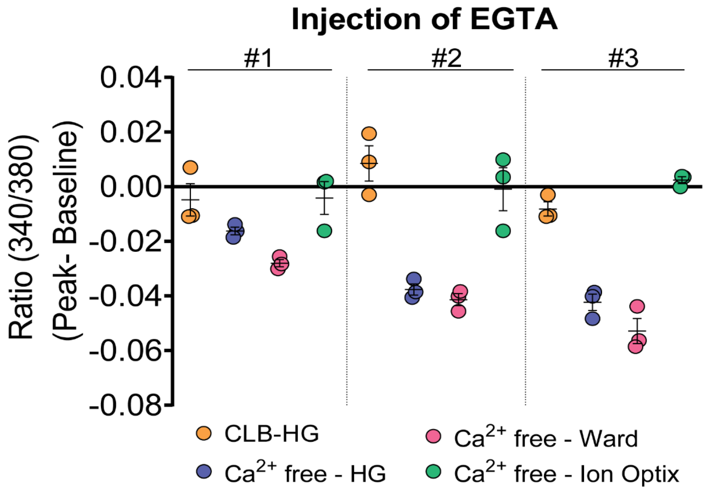

Figure S8: Addition of EGTA to Fura-2/AM and EGTA co-loaded cells chelates calcium in Ca^{2+}-free-HG and Ca^{2+}-free-Ward buffers. Cells co-loaded with Fura-2/AM and 10µM EGTA were stimulated with a further dose of 5.5 mM EGTA. Data shown as mean ± S.E.M for three independent experiments with three replicates in each experiment.
